# Regulatory Responses of Ectothermic Embryos to Predicted Heat Stresses Under Near‐Future Climate Change

**DOI:** 10.1111/mec.70456

**Published:** 2026-07-10

**Authors:** Reena H. Walker, Ishani Sinha, Nicolas Rochette, Thomas J. Sanger, Shane C. Campbell‐Staton

**Affiliations:** ^1^ Department of Ecology & Evolutionary Biology Princeton University Princeton New Jersey USA; ^2^ Department of Biology Loyola University Chicago Chicago Illinois USA

**Keywords:** climate change, development, heat stress, plasticity, thermal physiology

## Abstract

Heat stress can disrupt coordinated regulatory processes underpinning normal development, resulting in embryonic malformation and inviability. Embryonic buffering against thermal challenge may therefore play a crucial role in individual survival and population resilience under heat stress. Yet, the molecular mechanisms underpinning embryonic defence against ecologically‐meaningful heat stresses remain poorly understood. We investigated the regulatory responses of brown anole ( *Anolis sagrei* ) embryos to three patterns of heat stress we find will increase under near‐future climate warming. Across chronic, persistent, and acute heat treatments, embryos exhibited rapid transcriptome‐wide disruption followed by recovery toward normal developmental trajectories within 24 h. Despite this broad perturbation, a core regulatory module governing forebrain and craniofacial development remained strongly preserved across heat insults. Heat stress induced widespread transcriptional suppression alongside increased reliance of post‐transcriptional regulation and a dose‐dependent slowing of RNA flux, consistent with mitigation of proteotoxic stress. Concurrently, embryos rewired regulatory networks through recruitment of extramodular stress‐response genes and strengthening co‐expression within developmental pathways. Together, these results reveal a hierarchical regulatory strategy in which embryos buffer essential developmental programs while flexibly reconfiguring peripheral networks to manage cellular stress. This systems‐level resilience underscores the importance of early‐life adaptive regulatory plasticity in shaping organismal responses to climate warming.

## 1 Introduction

Environmental temperature is a universal pressure, constraining the limits of growth, performance, and fitness across all levels of biological hierarchy (Dell et al. 2011). Rising temperatures due to global warming, urban heat island effect, and the growing frequency and intensity of heat waves impose increasing physiological stresses on wildlife (Root et al. 2003; Oke 1973; Hansen et al. 2012). All living organisms rely on dynamic thermoregulatory mechanisms to buffer against heat stress and maintain homeostasis required for proper growth, survival, and reproduction (Angiletta 2009). The effectiveness of this adaptive plasticity depends on thermal dose (Stuhr et al. 2017; Niehaus et al. 2012; Ruel and Ayres 1999), evolutionary history (MacLean et al. 2016; Kellemann et al. 2012; Losos 2008), and organismal life stage (Wolf et al. 2023; Sales et al. 2021; Levy et al. 2015). For example, heat is a particularly potent teratogen during embryonic development (Edwards et al. 2003), during which heat stress can induce cell death and disrupt critical signalling pathways underpinning normal development, leading to numerous maladaptive biological consequences later in life (Sanger et al. 2021; Hansen 2007; Edwards 1998). Temperatures just 2°C–4°C outside the range required for the proper progression of normal morphogenesis can lead to craniofacial malformations, neural defects, reductions in brain size, behavioural abnormalities, and poor survivorship across vertebrate taxa, including mammals (Moretti et al. 2005; Webster and Edwards 1984), birds (Nilsen 1984; Ande and Wilson 1981), fish (Han et al. 2020; Erikson et al. 2006), and reptiles (Sanger et al. 2021; Sanger et al. 2018). Thus, while models of species' sensitivity to climate have been biased toward understanding adult responses to chronic thermal challenge, thermoregulatory insult during early development may significantly influence the health, viability and evolution of wildlife facing near‐future climate change (Dahlke et al. 2020; Levy et al. 2015).

Oviparous species, many of which display little or no parental care after egg deposition, may be particularly vulnerable to thermal insults during embryonic development. For many of these species, the embryonic thermal incubation environment is determined by maternal nest site selection (Angilletta et al. 2009; Reedy et al. 2012), leaving developing embryos at the mercy of external abiotic conditions at the site of egg deposition. Egg‐bound embryos must cope with daily temperature fluctuations, variable mean temperatures, prolonged periods of thermal stress, and acute heat shock exposure to survive in modern ecosystems (Sanger et al. 2018). With a limited ability to behaviorally buffer against these various forms of heat stress (Telemeco et al. 2016; Li et al. 2014; Du et al. 2011), adaptive regulatory plasticity—the ability to alter patterns of gene expression, gene–gene relationships (regulatory coherence), regulatory network structure, or post‐transcriptional dynamics to improve fitness in response to environmental challenge (West‐Eberhard 2003)—may play a critical role in the ability of egg‐bound embryonic life to maintain normal patterns of craniofacial development during thermal insult.

While specific and ordered transcriptional programs are required for proper developmental patterning, the expression of genes outside of those programs is more variable and can be differentially modulated in response to environmental cues (MacNeil and Walhout 2011). The cellular heat‐shock response is an evolutionarily well‐conserved protective process to mitigate the cellular stress induced by protein misfolding and aggregation under thermal challenge (Lindquist 1986). To mount this critical compensatory response, cells must significantly reorganize their regulatory program (Himanen and Sistonen 2019; de Nadal et al. 2011). Cellular heat‐shock response rapidly upregulates a suite of chaperones to assist protein folding and help clear damaged macromolecules while downregulating thousands of genes that regulate the cell cycle, transcription, and metabolism (Himanen and Sistonen 2019; Lepock 2003). If this heat‐shock response is insufficient to protect cells from the accumulation of degraded proteins, damaged DNA, and chromosomal aberrations, cells enter a state of programmed cell death (Lepock 2003). Under heat stress, embryonic cells must therefore navigate a trade‐off between maintenance of proper developmental trajectory and protection against heat‐induced threats to homeostasis (Marshall et al. 2020). However, though specific molecular chaperones, such as the heat shock proteins (HSPs), have been well studied across many species (Sørensen et al. 2003), we still know surprisingly little about the broader systems biology of embryonic response to heat stress, how developing cells calibrate investment in response to thermal stress that can vary in magnitude and exposure time, or the degree to which adaptive regulatory plasticity can protect sensitive cell signalling pathways required to maintain proper morphogenesis under the types of thermal challenges experienced in nature (Pottier et al. 2022; Nijhout 2002). Understanding the embryonic mechanisms that maintain patterns of normal development in the face of heat stress will provide critical information about heat resilience and potential targets of heat‐induced selection under climate change that are not readily visible at later adult stages. To fill this gap, we evaluated patterns of adaptive regulatory plasticity during embryonic development of the forebrain and face of the Cuban brown anole ( *Anolis sagrei* ), a widespread thermal generalist and burgeoning model system for vertebrate development under heat stress (Sanger et al. 2018, 2021; Gleason et al. 2023).

**FIGURE 1:**
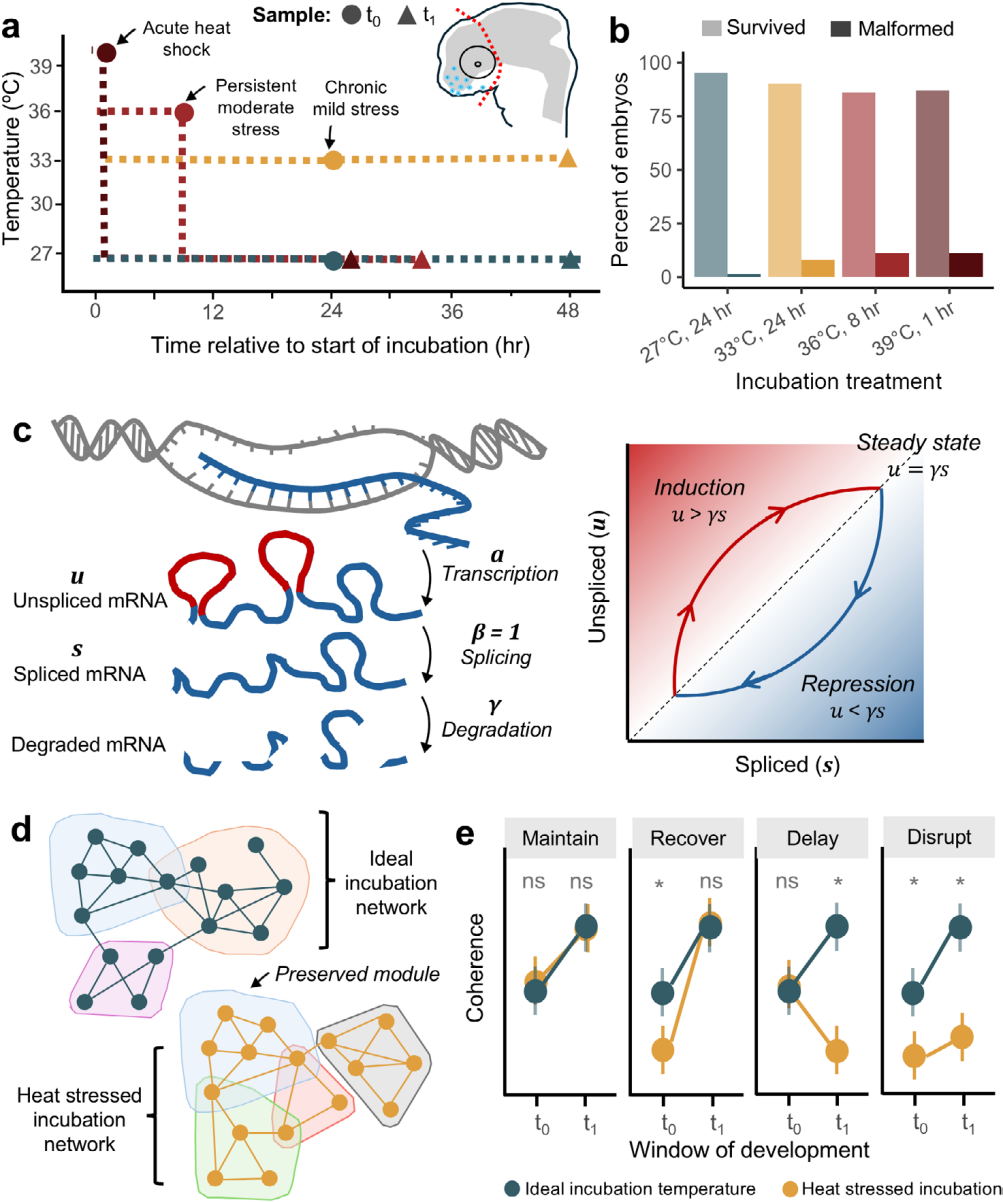
Incubation treatments and potential regulatory responses to thermal challenge. (a) We collected tissue from embryos across a control thermal treatment (blue) and three thermal stress treatments (yellow, red, dark red) immediately following thermal challenge (t_0_) and 24 h later (t_1_) to parse regulatory responses to stress and recovery. Thermal trajectories represented by lines; collection points represented by circles. Inset diagram illustrates the site of forebrain and face tissue collection (rostral of dotted red line). (b) Incubation treatments are stressful and are sufficient to reduce embryonic survival within 12 days after oviposition, while increasing likelihood of craniofacial malformations in *A. sagrei* embryos relative to the control treatment (blue). Data from Sanger et al. (2021). (c) Model of transcriptional dynamics illustrating the RNA progression cycle: Transcription (*a*), splicing (*β*), and degradation (*γ*) rates involved in production of unspliced (*u*) and spliced (*s*) mRNA products. Graph shows unspliced and spliced mRNA dynamics in response to step changes in α, “RNA flux”. Dashed line illustrates steady states for different values of transcription rates *α*, given by slope *γ*. Red line and shading represent levels of unspliced mRNA above that proportion, indicating increasing expression of a gene; blue line and shading represent decreasing expression of a gene. Adapted from La Manno et al. (2018). (d) Potential impacts of heat stress on gene regulatory networks. Circles represent network nodes (genes), lines represent connections (gene–gene correlations), polygons represent network modules (clusters of co‐expressed genes). Preserved modules (blue polygon) under heat stress are composed of the same nodes and connections as those in the ideal incubation treatment. (e) Classification of potential impacts of heat stress on coordinated gene–gene interactions (regulatory coherence). Maintained genes show no statistical difference (ns) in coherence between control (blue) and heat stressed (yellow) treatments. Recovered genes show significant divergence from the control (*) immediately following heat stress (t_0_) but return to the control baseline the following day (t_1_). Delayed genes show no difference from the control at t_0_ but significant divergence at t_1_. Disrupted genes are classified as those displaying significantly differences from control at both ends of the developmental window.

We investigated transcriptomic responses of the precursors to the forebrain and anterior skull. (telencephalon, craniofacial structure, eye, and neural crest cells, Figure 1a) to thermal challenges paralleling those experienced in natural nest sites within the species' native range. Morphogenesis of the brain and face is complex and heat sensitive: high incubation temperatures can derail proper formation of the skull (Sanger et al. 2021) and impair social and spatial cognition in individuals without morphological anomalies (Siviter et al. 2017; Abayarthna and Webb 2020), creating fitness consequences in ovo that persist through later life stages. To evaluate potential mechanisms protecting these processes from environmental heat stress, we exposed embryos collected at the time of oviposition to thermal treatments simulating persistent, chronic, and acute thermal insults that embryos currently experience in natural nest sites (Sanger et al. 2021) and that may become more common over the next 75–100 years with rising mean temperatures, heat waves, and more extreme thermal maxima (Root et al. 2003; Oke 1973; Hansen et al. 2012). The chosen thermal treatments are sufficiently challenging to reduce survivorship to 86%–87% and induce craniofacial malformations in 8%–11% of exposed embryos (Figure 1a,b; Sanger et al. 2021). To identify heat‐induced regulatory deviations from normal development, we compared each of the three heat treatments to a control treatment representing the congruent developmental timeframe in which embryos are expected to maintain regulatory homeostasis (constant 27°C) (Sanger et al. 2018, 2021). We compared developing forebrain and face transcriptomes among staged‐matched embryos to isolate the impact of experimental heat stress and identify transcriptional and post‐transcriptional responses to thermal challenge during development (Figure 1c–e).

## 2 Methods

### 2.1 Estimating Future Nest Temperatures

To test the prediction that thermal stress on oviparous embryonic life is likely to increase over the next century, we first quantified the proportion of the *A. sagrei* range where maximum daily ambient temperatures (2‐m above ground surface) exceeded safe nesting temperatures (> 33°C) in 20‐year intervals using publicly available historic (2010–2021) climate data and future (2021–2100) downscaled climate projections under a moderate warming scenario (GSM CMIP6, SSPs projection 370; Fick and Hijmans 2017; Roll et al. 2017; Caetano et al. 2022). We tested whether a significantly greater proportion of the species' range is likely to experience mean maximum ambient temperatures exceeding safe nesting thresholds in 2081–2100 compared to recent historic levels (2010–2021) using an equality of proportions test in R (v.4.5.0; R Core Team 2025).

Next, we estimated nest‐site microclimate conditions over the next century using the biophysical model, NicheMapper, to integrate a suite of environmental variables influencing radiation, convection, and conduction and estimate hourly temperatures at a 2.5° spatial and monthly temporal resolution (Kearney and Porter 2016; Kearney et al. 2014). We estimated the average hourly temperatures expected for loose soil at a depth of 2.5‐cm in 50% shaded sandy loam across the *A. sagrei* native range (Sanger et al. 2018, 2021; Fick and Hijmans 2017; Roll et al. 2017) (Table S1). We then tested if the number of hours per day that nest sites historically (2010–2021) experienced within the critical window of *A. sagrei* embryo thermal sensitivity (> 33°C) are likely to increase in the future (2081–2100) by fitting a generalized linear mixed effects model with nested month‐per‐cell random intercepts to control for spatiotemporal autocorrelation using the *glmm*t*MB* package (v.1.1.10; Brooks et al. 2017) in R.

### 2.2 Animal Husbandry

All animal care and handling procedures were approved by the Loyola University Chicago IACUC committee (protocol #1992). Detailed *Anolis* husbandry and egg incubation protocols are described in Sanger et al. (2008). Briefly, gravid females were collected from an invasive population in Coral Gabels, FL in 2021 and housed at Loyola University Chicago in groups of 4–6 individuals per cage. Cages were kept in 27°C incubators and were equipped with perches and a pot of moist vermiculite for females to lay their eggs. Lizards were fed crickets daily and misted five times per day with an automated system (Mist King, Jugle Hobbies Ltd.). Eggs laid within the first week after transport were not included in the study. We collected eggs between 09:00 and 10:00 hours each day and randomly assigned them to an incubation treatment.

### 2.3 Tissue Collection, RNA Extraction, and Sequencing for Transcriptomic Analyses

To evaluate regulatory mechanisms underpinning response to embryonic heat stress, we immediately transferred eggs to one of four incubation treatments: (1) control treatment (27°C, 24 h), (2) chronic mild stress (33°C, 24 h), (3) persistent moderate stress (36°C, 8 h), or (4) acute heat shock (40°C, 1 h). We dissected developing forebrain and face tissue from 10 stage‐matched embryos (Sanger stage 3/stage 4; Sanger et al. 2008) immediately following each treatment (t_0_). An additional set of eggs from the control, persistent moderate stress, and acute heat shock treatments were then incubated at 27°C to evaluate potential mechanisms of developmental recovery after heat stress. Lastly, a set of eggs from the chronic mild stress treatment were maintained at 33°C to evaluate the impacts of long‐term exposure to low‐dose heat. After 24 h, we collected telencephalon tissue from 10 stage‐matched embryos (Sanger stage 4/5) from each treatment group (t_1_) (Figure 1a). This experimental design allows us to parse mechanisms of initial stress response from those of potential developmental recovery.

We collected tissue for subsequent analyses by dissecting embryos at Loyola University Chicago while eggs were submerged in sterile PBS. We made an incision around the posterior margin of the eye using a 0.125 mm tungsten needle to collect developing brain and face tissue. Tissue was immediately flash frozen in liquid nitrogen and stored at −80°C until shipment on dry ice to Princeton University for RNA extraction.

Total RNA was extracted from 80 tissue samples using Qiagen RNeasy Mini Kits and quantified using a Qubit fluorometer (Life Technologies). Messenger RNA libraries were prepared at the Genomics Core Facility at Princeton University using the 3′mRNA SMART‐seq Library Prep Kit and sequenced for 150 base pair single‐end reads on the NovaSeq S1 100 nt Flowcell v1.5, resulting in an average of ~11 M ± 3.8 reads per sample. Raw expression data were trimmed to remove low‐quality reads and adapter sequences with Cutadapt (Martin 2011). Reads were aligned to *A. carolinensis* reference genome using STAR (Dobin et al. 2013) under relaxed alignment score parameters (Lscore = 0.3). We filtered the uniquely mapped read count data to exclude genes with an average ≤ 10 reads across samples, resulting in a final telencephalon transcriptome composed of 9626 genes.

### 2.4 Statistical Analyses of Individual Gene Expression

We first evaluated overall patterns of gene expression associated with embryonic development and heat stress with a principal component analysis (PCA). After normalizing raw read counts by library size (cpm function in edgeR package, v.4.2.1; Robinson et al. 2010), we summarized expression for each sample using the prcomp function in R. We fit separate linear regression models for the first two principal components on each day of development to test transcriptome‐level differences in developing telencephalon tissue among heat stress treatments. We quantified divergence from normal patterns of expression on each day of development as the Euclidean distance of the mean PC coordinate of samples for each treatment from the mean PC coordinate of the control treatment.

Next, we used the edgeR package (Robinson et al. 2010) to estimate log‐fold differential expression between control and thermal stress treatments, immediately after thermal challenge (t_0_) and 24 h after thermal challenge (t_1_) for each incubation condition. We identified a core set of up‐ and down‐regulated genes under embryonic heat stress as those differentially expressed across all heat stress treatments. We evaluated driver terms for biological processes associated with common up‐ and down‐regulated genes across heat treatments using an unordered query in gprofiler2 (v.0.2.3; Kolberg et al. 2020).

### 2.5 Statistical Analyses of Post‐Transcriptional Regulatory Divergence

We evaluated the contributions of post‐transcriptional modification in driving differences in gene expression under heat stress using exon‐intron split analysis (EISA; Gaidatzis, Burger, et al. 2015; Gaidatzis, Lerch, et al. 2015) to measure changes in mature RNA and pre‐mRNA reads across the different heat treatments relative to those observed in ideal incubation conditions (Figure 1c). Only transcripts that mapped to unique positions on the genome were considered for EISA. Exon/intron counts were enumerated using qCount from QuasR (Gaidatzis, Burger, et al. 2015; Gaidatzis, Lerch, et al. 2015) in R. Following Gaidatzis, Burger, et al. (2015); Gaidatzis, Lerch, et al. (2015), we linearly scaled intronic and exonic counts to mean library size (sum of all intronic or exonic counts, respectively). Quantifiable genes (*x*) were selected as those with counts where log2(*x* + 8) > 5. This conservative gene selection criterion resulted in a range of 191–244 quantifiable genes across the eight treatment‐days. We used the quasi‐likelihood f‐test from the edgeR package (Robinson et al. 2010) to model the statistical significance of differential post‐transcriptional regulation. We used NBCI and Panther databases to investigate the function of post‐transcriptionally regulated genes. We quantified differences in spliced and unspliced gene abundance between control and heat‐stressed embryos on each day of development by fitting linear mixed effects models with per‐gene random intercepts with the lme4 package (v.1.1‐35.5; Bates et al. 2015).

### 2.6 Statistical Analyses of Modular Architecture Within the Transcriptome

We used weighted gene correlation network analysis (WGCNA, v.1.73; Langfelder and Horvath 2008) to identify de novo co‐expression networks associated with developmental progression of the telencephalon transcriptome using t_0_ and t_1_ samples from each incubation treatment. After log‐transforming and normalizing raw count data by library size, we used Pearson correlations to identify and remove outlier samples. Next, we computed an adjacency matrix and used a soft‐thresholding approach (power = 12) to approximate a scale‐free topological network. We then used topological overlap to create a cluster dendrogram based on hierarchical clustering. We used branches of the resulting cluster tree to identify regulatory modules via the dynamic tree cutting method, merging highly correlated modules (*R* ^2^ = 0.75) for downstream analyses (Langfelder and Horvath 2008). To identify regulatory modules associated with development, we first summarized module‐level expression using a PCA of gene expression for each module with the *blockwiseModules* function in WCGNA (Langfelder and Horvath 2008). We summarized regulatory variation for each module identified using PC1 (referred to as the module eigengene; Langfelder and Horvath 2008). We then tested for significant eigengene associations with this window of development (i.e., t_0_ vs. t_1_ expression) by Pearson correlation and the Student's asymptotic test using the cor and *corPvalueS*t*uden*t functions in WGCNA (Langfelder and Horvath 2008). We used an ordered query ranked by genes' module membership in gprofiler2 (Kolberg et al. 2020) to identify gene ontology categories enriched in development‐associated modules from each incubation treatment. Finally, we tested for evidence of disproportionate representation and centrality among an a priori list of genes with known functional relationships with embryonic forebrain and craniofacial morphogenenesis in association with focal development‐associated module (O'Leary et al. 2007; Minoux and Rijli 2010; Trainor and Krumlauf 2000). Using the R package biomaRt's *useMar*t*()* and *ge*t*BM()* functions to link ensembl gene identifiers to gene names (v.2.60.1; Durinck et al. 2005, 2009), we identified which of the 34 *apriori* candidate genes were found in our anole forebrain and anterior skull transcriptome and (1) conducted a one‐sided Fisher's exact test for count data to tested whether the candidates were significantly more likely to fall in the development‐associated module than elsewhere, (2) conducted a Welch's two sample *t*‐test to evaluate whether the candidate genes' mean module connectivity was greater than the module mean, and (3) quantified candidate genes' percentile of connectivity within the development‐associated module relative to others in the network.

We next tested whether the regulatory architecture underpinning normal change in this window of development was preserved under heat stress with the composite module preservation summary statistics, *Z*_summary_ and medianRank, using the modulePreservation function in WGCNA (Langfelder and Horvath 2008, 2012; Langfelder et al. 2011) (Figure 1d). *Z*_summary_ is a quantitative measure of network similarity that can be used to determine whether module nodes in a reference network remain highly connected and maintain the same pattern of connectivity in a test network (Langfelder et al. 2011). Values of *Z* > 10 provide strong evidence that the module is preserved in the test network, values 2 < *Z* < 10 indicate weak to moderate evidence of preservation, and values 0 < *Z* < 2 suggest no evidence of preservation (Langfelder et al. 2011). *Z*‐scores are established by randomly permuting module assignments in the test data to estimate a null distribution of the preservation statistics and determine whether the observed value of a preservation statistic is higher than expected by chance (Langfelder et al. 2011). The medianRank statistic is useful for comparing relative preservation among multiple modules and is much less dependent on module size than Z statistics (Langfelder et al. 2011).

### 2.7 Statistical Analyses of Regulatory Coherence

We evaluated the impact of heat stress on regulatory coherence using correlation by individual level product (CILP; Lea et al. 2019). To parse regulatory impacts of stress response, potential recovery, and overall developmental trajectory, we contrasted coherence (i) between ideal and stressed conditions immediately after thermal challenge (t_0_), (ii) between ideal and stressed conditions 24 h after thermal challenge (t_1_), and (iii) between days of development (t_0_, t_1_) for each incubation condition. We scaled and centered read counts and calculated the Spearman correlation coefficient for each gene–gene pair within each treatment‐day, then used ordinary least squares regression to contrast the strength and direction of gene–gene correlations between classes (Lea et al. 2019). We identified gene–gene connections critical for this window of development as those with significant change in correlation between t_0_ and t_1_ under ideal incubation conditions (CILP: *p* < 0.05). We classified these gene–gene connections as (i) maintained when correlation was equivalent (not statistically different, *p* > 0.05) in control and stressed conditions on both t_0_ and t_1_, (ii) *recovered* when correlation differed between control and stressed conditions on t_0_ but not on t_1_, (iii) *delayed* when correlation was equivalent between control and stressed conditions on t_0_ but differed on t_1_, or (iv) *disrup*t*ed* when correlation differed from control both on t_0_ and t_1_ (Figure 1e). We used gprofiler2 (Kolberg et al. 2020) to identify gene ontology categories enriched in genes–gene relationships maintained under thermal challenge via an ordered query sorted by the number of times genes were observed in maintained pairwise relationships.

We tested whether new gene–gene relationships emerged under heat stress to support development by evaluating pairwise relationships between genes within the module associated with normal development and genes in the rest of the network. We randomly selected 1,000,000 of these gene pairs, contrasted coherence under heat stress on t_0_ and t_1_ within each treatment using CILP (Lea et al. 2019), and classified pairwise relationships as (i) *gained*, when coexpression was not observed (Spearman correlation test, *p* > 0.05) under control conditions but significant under heat stress, (ii) lost, when genes were significantly coexpressed under control but not under heat stress, or (iii) *preserved*, when a gene pair was significantly coexpressed under both normal and stressed conditions. We used gprofiler2 (Kolberg et al. 2020) to identify biological processes associated with relationships gained under heat stress using an ordered query sorted by the number of instances each extramodular gene appeared in significant (*p* < 0.05) pairwise relationships.

## 3 Results and Discussion

### 3.1 Embryonic Stress Under Near‐Future Climate Change Scenarios

**FIGURE 2:**
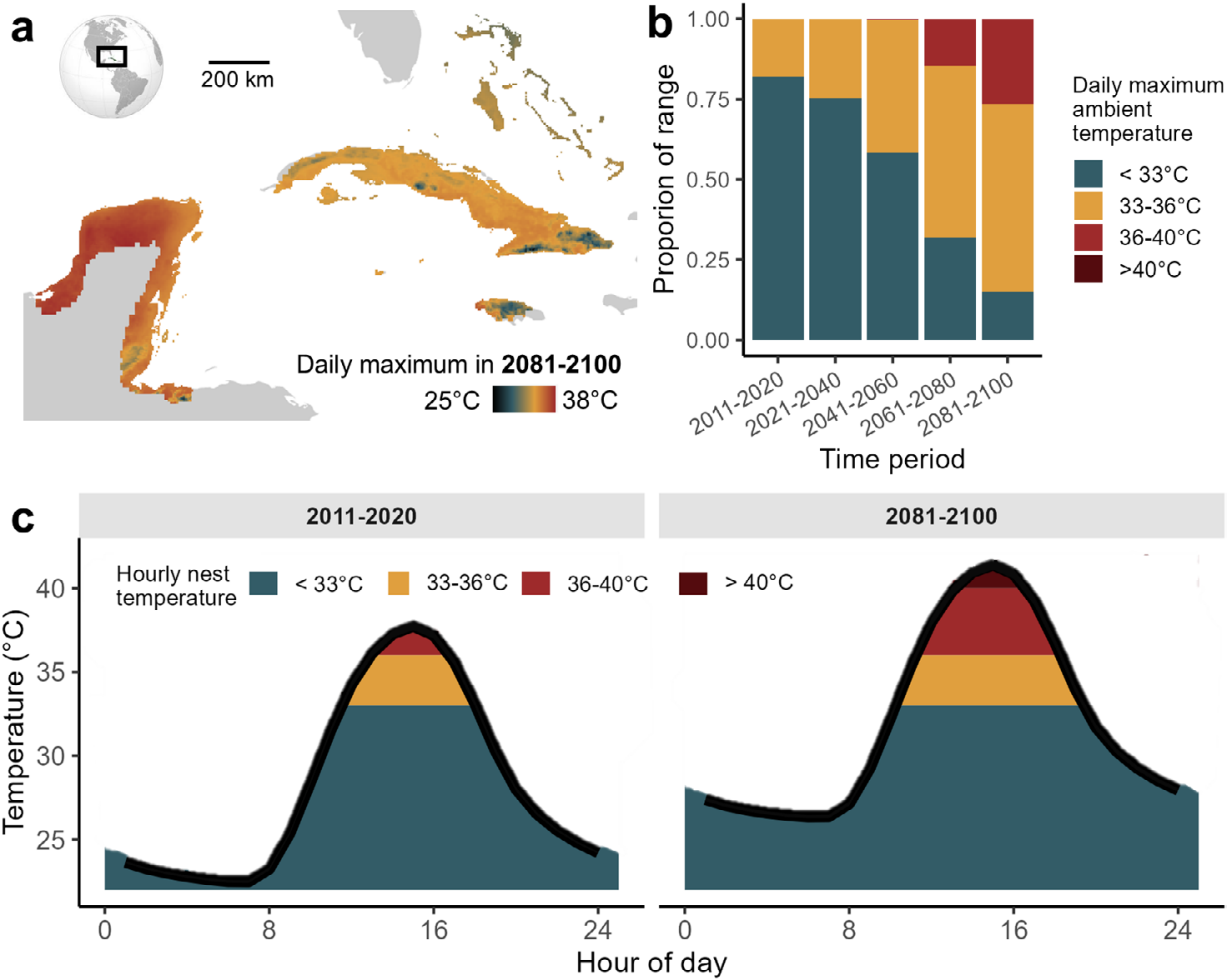
Temperatures inducing embryonic heat stress are becoming more common across *A. sagrei* 's native range. (a) The average daily maximum temperature will exceed 33°C across most of brown anoles' native range by the end of the century under a moderate warming scenario (SSPs370). Map lines delineate study areas and do not necessarily depict accepted national boundaries. (b) While < 25% of *A. sagrei* 's native range experienced daily maximum ambient temperatures exceeding safe nesting temperatures over the last decade, the average day in > 85% of the range will reach ambient temperatures sufficient to reduce embryonic survival and induce craniofacial malformations in the by 2100. (c) Controlling for spatiotemporal variation across the range, three additional hours per day will exceed 33°C in *A. sagrei* nests microclimates (2.5 cm soil depth, 50% sun exposure) by the end of the century.

Understanding organisms' tolerance to both chronic and acute heat stress at their most vulnerable life stage is critical for predicting impacts of climate change on population dynamics, species' distributions, and evolutionary trajectories in the coming century (Levy et al. 2015; Pilakouta et al. 2024; Beever et al. 2010; Chen et al. 2018). Therefore, we first evaluated how heat stress scenarios experienced in *A. sagrei* nest sites across their range may change over the coming century. We find that *A. sagrei* embryos are likely to confront significantly more challenging thermal conditions during embryonic development by the year 2100. Both daily maximum ambient temperatures and simulated nest site temperatures will significantly increase across the *A. sagrei* native range compared to historic conditions (Figure 2). While mean annual ambient daily maximums exceeded safe incubation temperature (33°C; Sanger et al. 2021) in just 18% of the species' native range in the last decade (2011–2020), over 85% of the native range will experience ambient temperatures dangerous to embryonic development by the end of the century under the SSP370 moderate warming scenario (equality of proportions test: *X* ^2^ = 11,964, df = 1, *p* < 0.001) (Figure 2a,b). By 2100, the average *A. sagrei* nest site (2.5 cm depth, 50% shade coverage) will experience over three additional hours per day at temperatures exceeding safe incubation thresholds compared to recent historic conditions (GLMM with nested per‐cell/month random intercepts: *β*_2081‐2011_ = 3.1, SE = 0.003, *p* < 0.001; Figure 2c). Over an average day in 2081–2100, eggs in typical nest sites will experience temperatures exceeding 33°C for 10 h, 36°C for 7 h, and 40°C for 3 h. Previous work has shown that anole embryos display an imperfect ability to buffer against stress induced by these thermal challenges (Sanger et al. 2018, 2021). Laboratory‐based heat challenge experiments simulating these conditions reduce embryo survivorship by > 10% and induce a four‐fold increase in developmental malformations compared to embryos developing under control incubation conditions (27°C) (Sanger et al. 2018, 2021) (Figure 1b). Consequently, climatic conditions by the end of the century may substantially reduce *A. sagrei* 's reproductive success and juvenile fitness throughout much of their native range. Under future warming, the ability of *A. sagrei* to colonize new areas (naturally or by human‐mediated transport) may compensate for declines in habitat suitability across their native range. Future studies should investigate whether the species' numerous successful invasive populations across the Caribbean, North America, and Pacific Islands (Kolbe et al. 2004; Kolbe et al. 2007) offer *A. sagrei* refuge from climate‐induced declines in fitness and if *A. sagrei* 's distribution will shift out of its native range toward more thermally favourable regions as mean temperatures increase under future warming.

### 3.2 Patterns of Divergent Heat‐Induced Single Gene Regulation Under Diverse Heat Stress Scenarios

**FIGURE 3:**
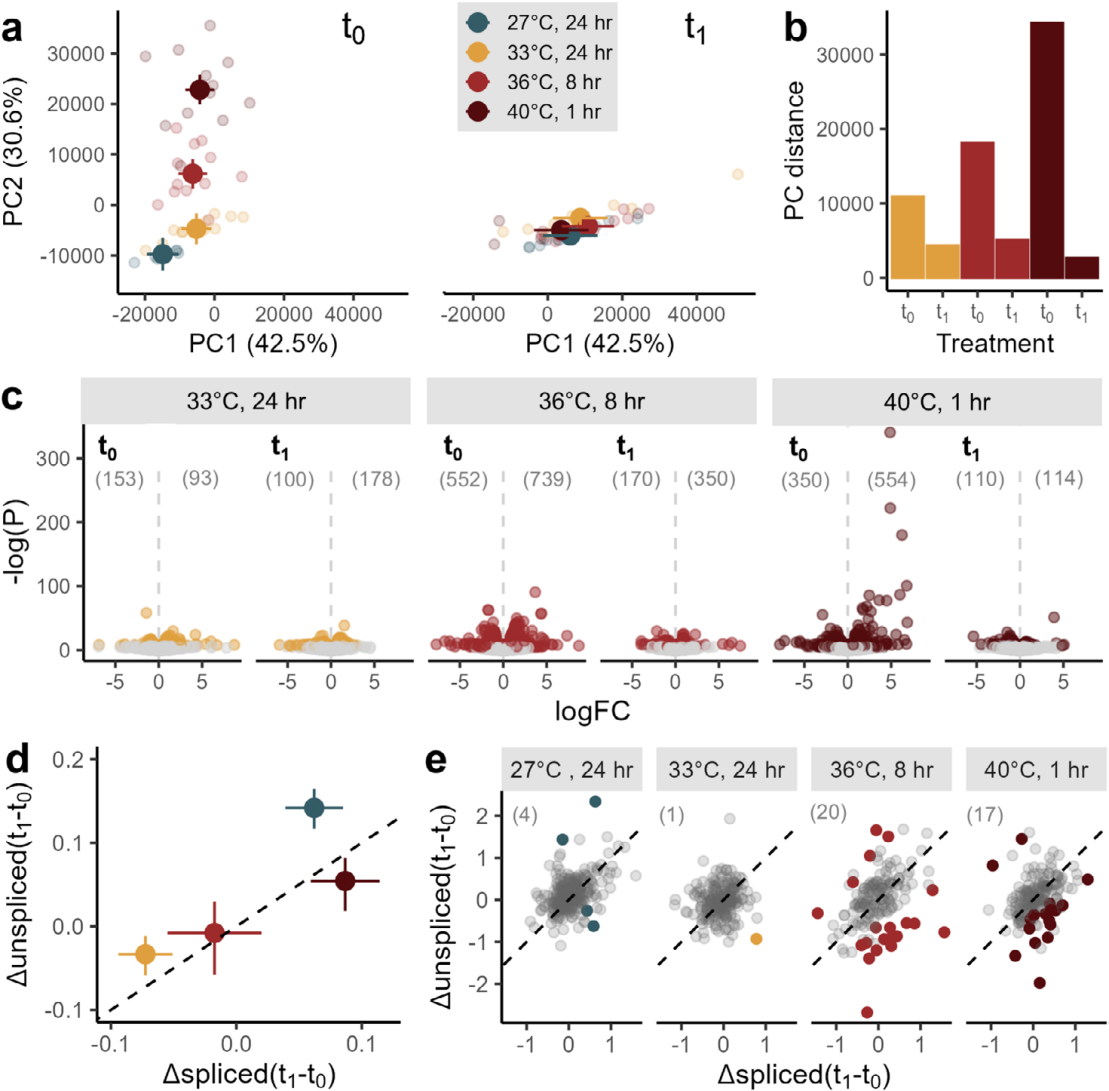
Whole‐transcriptome disruption, recovery, and repression in developing brain and face tissue after embryonic heat stress. (a) Principal component analysis summarizing whole‐transcriptome gene expression of brain and face tissue developing under control conditions (blue; consistent 27°C) versus three heat stress scenarios. Small points represent sample‐level PCs; large points are mean treatment PCs; bars illustrate 95% confidence intervals from linear regression models. (b) Euclidean distance of mean gene expression immediately following heat stress (t_0_) and 24 h later (t_1_) from expression under ideal conditions. Immediately after heat exposures, patterns of embryonic gene expression diverged significantly from normal across treatments but returned to normal after 24 h of recovery. (c) Volcano plots of significance (‘‐log(P)’) against the magnitude of difference in gene expression (‘logFC’) under each treatment compared to normal on each day of development. Coloured points represent differentially expressed genes. Annotations denote the number of up‐ and down‐regulated genes under each treatment‐day. (d) RNA flux across incubation treatments. Points represent mean, bars represent standard error. Dashed line illustrates steady states for different values of transcription rates *α*, given by slope *γ*. Levels of unspliced mRNA above and below steady state indicates gene induction and repression, respectively. (e) Change in spliced and unspliced mRNA abundance over this window of development across treatments and days of development. Coloured points represent post‐transcriptionally regulated genes under each incubation treatment; dashed line represents steady state.

Embryos that are able to develop normally despite significantly elevated nest temperatures may employ plastic, heat‐induced physiological responses to maintain normal developmental trajectories in the face of such thermal challenge. Indeed, we find exposure to heat stress results in whole‐sale regulatory disruption and dose‐dependent recovery toward normal expression in embryonic telencephalon tissue in the early stages of morphogenesis (Figure 3a,b). Expression in the developing forebrain and face tissue immediately following heat stress significantly diverged from patterns quantified under control incubation conditions (OLS regression models fit separately for PC1 and PC2 on t_0_: *p* < 0.03 across models/treatments; Tables S2 and S3). Overall expression in embryos exposed to persistent moderate heat stress and acute heat shock largely recovered after 24 h of recovery at control incubation temperatures (OLS regression models fit separately for PC1 and PC2 on t_1_: *p* > 0.08 across models/treatments; Table S3). Significant regulatory divergence after thermal challenge followed by the return of overall expression to resemble control conditions within 24 h after exposure support the hypothesis that regulatory responses after heat stress represent embryonic adaptive plasticity to defend systemic homeostasis against thermal challenge and maintain normal developmental trajectories. On the second day of incubation under chronic mild stress conditions, patterns of gene expression in heat stressed embryos continued to significantly diverge from control levels (OLS for PC2 on t_1_: *p* = 0.002; Table S3). Though we find signatures of recovery after transient heat treatments, return of normal physiological phenotypes lag behind the gene expression recovery (Whitehead 2012), and even short‐term perturbation of developmental processes can impact hatchling or adult fitness via depletion of energetic reserves by elevated metabolic rate (Sun et al. 2015), epigenetic modification that shift adult responses to temperature (Jonsson et al. 2022), or disruption of gene expression in critical signalling pathways (Sanger et al. 2021).

To quantify embryonic regulatory response to heat stress at the single gene level, we directly compared patterns of expression between control and heat stress treatments (Figure 3c). Immediately following heat stress (t_0_), 76 genes (7.6% of 1003 genes) were universally upregulated and 25 genes (3.2% of 773 genes) were universally downregulated across treatments (Figure S1). 24 h later (t_1_), fewer genes were differentially expressed across thermal stress treatments compared to the control (Figure 3c). Of these genes, 31 (6.5% of 485 genes) were universally upregulated across treatments and 5 (1.5% of 328 genes) were universally downregulated (Figure S1). This shared set of differentially expressed genes across thermal stress treatments is associated with core mechanisms by which cells compensate for damage under heat stress (Table S4). Heat stress depolymerizes microtubules, which in turn undermines the intracellular transport and cell division processes that depend on cellular structural integrity (Coss and Linnemans 1996). Cells resorb cilia under thermal challenge, diminishing the embryo's ability to regulate key extracellular signalling systems underlying neurogenesis (Prodromou et al. 2012). Protein–protein interactions are critical mechanisms by which cells compensate for protein unfolding, misfolding, and degradation in hyperthermic conditions (Roti 2007). The upregulation of genes at t_0_ associated microtubule movement (GO:0007017, GO:0007018), cilium assembly (GO:0044458, GO:0044782, GO:0003351), and protein binding (GO:0005515) immediately following heat stress is consistent with a stress mitigation response through maintenance of the Hedgehog signalling pathway, which is critical for telencephalon differentiation (Sanger et al. 2021; Prodromou et al. 2012). In concert, the downregulation of genes associated with transcription at t_0_ (GO:0005634, GO:0032993, GO:0042393) suggests rapid transcriptional suppression under thermal stress, a shared strategy across taxa to mitigate cellular damage via proteotoxicity (Vihervaara et al. 2018). On t_1_, thermally stressed embryonic brain and face cells displayed upregulation of genes primarily associated with posttranscriptional regulation (GO:0071005) and downregulation of genes and pathways related to management of cellular stress (GO:1900225, GO:0140545), suggesting a pattern of physiological recovery and return toward homeostasis.

### 3.3 Posttranscriptional Response to Embryonic Heat Stress

The downregulation of genes associated with transcription and upregulation of genes associated with posttranscriptional mechanisms in developing forebrain and face suggests that dynamic adjustments to the timing and magnitude of flux through the RNA progression cycle—transcription of premature mRNAs (*a*), splicing of premature mRNAs to produce mature mRNA (ß), and degradation of mature mRNA (*y*)—which we will refer to as ‘RNA flux’ (La Manno et al. 2018), may be an important mechanism of adaptive temperature‐dependent regulatory plasticity in the face of embryonic heat stress (Figure 1c). To test this hypothesis, we conducted exon‐intron split analysis (Gaidatzis, Burger, et al. 2015; Gaidatzis, Lerch, et al. 2015) to compare time‐dependent changes in production of mature mRNA (spliced) and pre‐mRNA (unspliced) products, respectively. Without a compensatory mechanism to maintain or decrease rates of RNA flux in embryos exposed to heat stress, we should observe increased rates of enzymatic activity and a concordant increase in RNA flux which exposes cells to a higher risk of proteotoxicity from an increased rate of misfolded protein production (La Manno et al. 2018; Beckski and Rahaman 2022; Tye et al. 2019). Instead, we find a general slowing of RNA flux under heat stressed conditions (Figure 3d), suggesting embryos rely on adaptive plasticity under thermal challenge that represses rates of RNA flux to mitigate heat‐induced accumulation of proteotoxic stress. Longer durations of heat exposure correspond with greater slowing of RNA flux such that the chronic mild stress treatment displayed the greatest reduction in RNA flux relative to control incubation temperatures, while the acute heat shock treatment shows the least. While acute exposures to high heat can trigger the cellular heat shock response and rapidly restore proteostasis post‐insult, protein maintenance under prolonged exposure to mild or moderate thermal stress presents a continuous challenge where even modest increases in protein misfolding accumulate over time (Pessa et al. 2024). If cumulative thermal exposure imposes stronger constraints on RNA metabolism than short‐lived extreme temperatures on embryonic development, chronic warming may pose a greater challenge to developmental homeostasis than acute thermal insults. As climate change manifests through elevated mean temperatures, warmer nights, and longer periods of sustained heat (IPCC 2021), constant transcriptional repression to avoid proteotoxicity may also suppress embryonic rate of development, though further work is needed to explicitly test this hypothesis.

At the individual gene‐level, we find posttranscriptional contributions to changes to RNA steady‐states are more common under persistent moderate stress and heat shock treatments than in the control and chronic incubation conditions (Figure 3e). Under control conditions, 4 genes associated with early brain development (*gadl1*, *mid1*) and mechanisms regulating RNA flux (*hnrnpl*, *gtf2e2*) are post‐transcriptionally regulated over this window of development (Table S5). None of these genes remained post‐transcriptionally affected under chronic mild heat stress, though post‐transcriptional regulation of *mid1* was preserved under both persistent moderate stress and heat shock treatments. These heat treatments were also associated with a marked rise in post‐transcriptional regulation relative to control: 20 genes under persistent moderate stress and 17 under heat shock display significant post‐transcriptional effects over this developmental window and are associated with brain development (e.g., *sat1, gpm6b, rbp1*), mechanisms regulating RNA flux (e.g., *wbp4, hnrnpl, isy1*), and cellular stress responses (e.g., *qrich1*, *tial1*, *ip6k2*) (Table S5). These enriched post‐transcriptional changes in steady‐state RNA levels under heat stress, together with evidence of dose‐dependent slowing of RNA flux across heat treatments, suggest that posttranscriptional mechanisms play an important role in buffering the impacts of medium‐ and short‐duration thermal challenge on genes whose expression is essential for development. This result aligns with other studies reporting the importance of posttranscriptional mechanisms in rapid response and recovery as the first line of defence against environmental stressors (Hernández‐Elvira and Sunnerhagen 2022).

### 3.4 Heat‐Induced Alterations of Regulatory Architecture Across the Transcriptome

**FIGURE 4:**
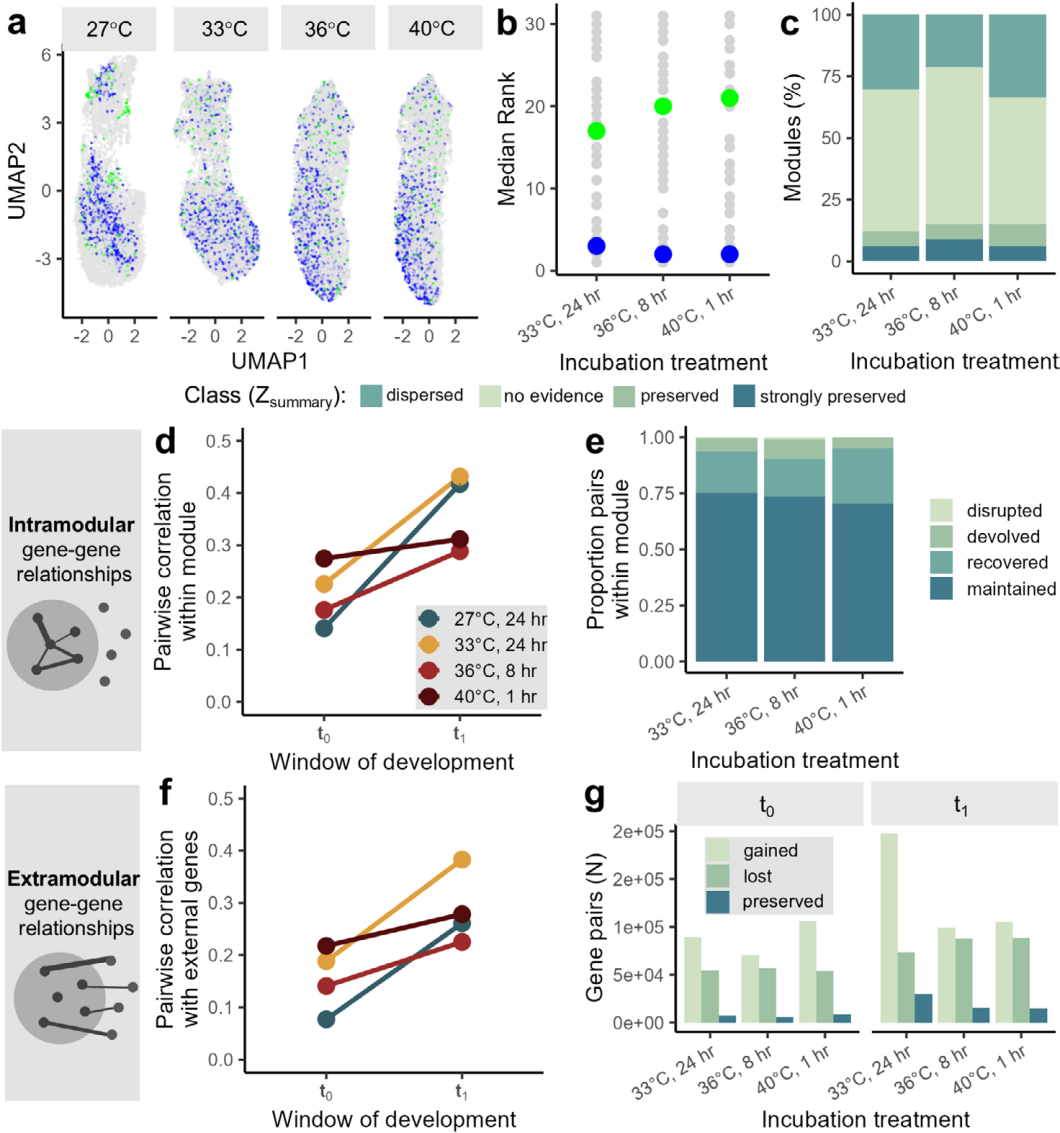
Thermal challenge rewires regulatory networks to support normal forebrain and face development. (a) Uniform manifold approximation and projection (UMAP) visualization of co‐expression networks across heat treatments. Points represent genes, adjacency approximates strength of co‐expression relationship; blue identifies nodes in the normal development module (*N* = 1087); green identifies nodes in a representative module that is not as well preserved (*N* = 303). (b) The development module (blue) is among the best preserved among treatments. A representative, poorly preserved module. Module (green; *medianRank*, range 17–21 across treatments) is highlighted for comparison. (c) Proportion of modules underpinning developmental trajectory (t_0‐1_) under ideal conditions (*N* = 30) in each preservation classification across heat treatments. The module associated with normal development was among the 10% (3/30) of preserved or strongly preserved co‐expression modules under heat stress. (d, f) Mean coherence among gene pairs in the module associated with normal development (*N* = 588,070) and among a sample of intra‐and extramodular pairs (*N* = 10^6^) immediately following heat stress (t_0_) and 24 h later (t_1_). Points show group means; SE bars subsumed by points; lines illustrate developmental trajectory (t_0‐1_). (e) Proportion of gene pairs within the development‐associated module that are maintained, recovered, devolved, and disrupted under each heat treatment. (g) Number of intra‐ and extramodular gene relationships gained (non‐significant normally, significant under heat stress), lost (significant normally, non‐significant under heat stress), and preserved (significant normally and under heat stress).

To further explore how embryos may defend developmental transcriptional programs against thermal challenge, we next tested whether and how gene regulatory networks formed over this window of development are perturbed under heat stress. Underlying the strong overall divergence in gene expression between control and heat stress treatments, embryos defended core regulatory programs required for differentiation and growth during the development window. A single regulatory module of 1087 genes was significantly associated with progression of neural development over the 48‐h experimental period under control incubation conditions (the ‘development module’; Pearson correlation and student asymptotic *p*‐value: cor = 0.56, *p* = 0.009; Table S6). This module was enriched for biological processes associated with glial cell differentiation (GO: 0010001, *p* < 0.001) and was significantly enriched with *apriori* candidate genes that regulate forebrain and craniofacial morphogenesis (*sox9, sox10, emx2, bmp7, dlx2*; One‐sided Fisher's exact test: odds‐ratio = 7.9, *p* = 0.003), three of which (*sox9*, *dlx2*, *bmp7*) were modular hub genes (i.e., had stronger intramodular connection than 97%–87% of the network) (Table S6; Trainor and Krumlauf 2000; Minoux and Rijli 2010). The development module was strongly preserved (*Z*_summary_ > 10, *medianRank* < 3) across heat treatments, despite pervasive regulatory rewiring over the rest of the transcriptome (Figure 4a–c). Of the 30 modules that compose the overall regulatory architecture of the tissue, 16–20 displayed no evidence of preservation under heat stress treatments while 7–11 modules displayed greater dispersion under heat stress than expected by chance (*Z*_summary_ range = −3.997, −2.012; Figure 4c). Instead, new co‐expression modules emerged in response to heat stress, enriched for processes associated with heat shock proteins (e.g., GO:0031072), protein folding (e.g., GO:0006457) and cell metabolism (e.g., GO:0009057) (Tables S7 and S8; Figure S2). Together, these results support the hypothesis that embryonic forebrain and face cells substantially invest in the defence of critical regulatory interactions required for development, potentially by mounting a strong stress response outside of those core transcriptional programs.

Similarly, we observe that genes within the development module strengthened intramodular co‐regulatory dynamics and formed novel significant extramodular co‐regulatory interactions under heat stress (Figure 4d–g). If embryonic forebrain and face cells adaptively defend developmental processes under heat stress, the strength of gene–gene relationships within the development module should remain consistent under thermal challenge and cells should maintain significant gene–gene relationships over this window of development. Indeed, we observe that intramodular coherence, (the mean strength of gene–gene relationships within the development module) was even more pronounced immediately following heat stress across all treatments than under control conditions (OLS regression: *β*(*chronic mild*) = 0.08 ± 0.006, *p* < 0.001; *β*(*persistent moderate*) = 0.03 ± 0.006, *p* < 0.001; *β(heat shock)* = 0.13 ± 0.006, *p* < 0.001), but was generally weaker than that observed in control embryos the following day (OLS regression: *β*(*chronic mild*) = 0.01 ± 0.005, *p* < 0.001; *β*(*persistent moderate*) = −0.13 ± 0.006, *p* < 0.001; *β*(*heat shock*) = −0.11 ± 0.006, *p* < 0.001) (Figure 4d, Table S9). Gene pairs that significantly change coherence over this window of development under control conditions (7%, 41,810/588,070, of intramodular pairs) largely maintained or recovered their patterns of coherence across heat treatments (i.e., correlation in expression between genes did not significantly differ from normal by t_1_; Figure 1e) [chronic mild stress: 93.7% (39,190/41810); persistent moderate stress: 90.3% (37,760/41810); heat shock: 95.1% (39,766/41810)] (Figure 4e). Of the maintained relationships, 46.0% (18,365/39,908) are common across heat treatments and enriched for loci associated with morphogenesis, neural development, and mitosis (e.g., GO:0007417, GO:0048731, GO:0007275, GO:0051960, GO:0009653) (Figure S3, Table S10). Conversely, only 2.8% (200/7093) of delayed or disrupted intramodular relationships are the same across heat treatments (Figure S3), suggesting the breakdown of coherence among intramodular gene‐pairs was idiosyncratic across thermal doses which is expected as a result of nonadaptive or maladaptive heat induced plasticity.

Stronger co‐expression and maintenance of significant gene–gene relationships in the development module under thermal challenge was supported by the recruitment of extramodular, stress‐response genes. Heat stress increased the mean strength of pairwise relationships between the development module and extramodular genes by more than twice the magnitude expected under ideal incubation conditions immediately following heat stress (mean ± SE Pearson correlation on t_0_ among 10^6^ random gene–gene pairs: control = 0.06 ± 0.064; chronic mild = 0.17 ± 0.003; persistent moderate = 0.12 ± 0.003; heat shock = 0.19 ± 0.003) (Figure 4f). Coherence among intra‐extramodular gene pairs continued to be significantly stronger relative to normal for the chronic mild and heat shock treatments after 24 h of recovery (mean ± SE Pearson correlation: control = 0.21 ± 0.003; chronic mild = 0.36 ± 0.003; heat shock = 0.27 ± 0.003) but was slightly weaker in embryos recovering from persistent moderate stress (0.19 ± 0.003) (Table S11). New relationships between genes in the development module emerged with genes across the transcriptome under heat stress (Figure 4g). Under control incubation conditions, 6.2% (62,111/10^6^) of randomly sampled intra‐extramodular gene pairs were coexpressed at t_0_ and 10.3% (10,3128/10^6^) were coexpressed at t_1_. Heat stressed embryos increased the number of extramodular relationships on both days of development [t_0_: chronic mild = 9.7% (96,804/10^6^), persistent moderate = 7.6% (75,810/10^6^), heat shock = 11.5% (114,668/10^6^); t_1_: chronic mild = 22.8% (227,598/10^6^), persistent moderate = 11.4% (114,712/10^6^), heat shock = 11.9% (119,391/10^6^)] compared to control conditions. Across heat treatments at t_0_, commonly gained relationships were composed of extramodular genes related to the negative regulation of cellular processes (GO:0048519, GO:0010948, GO:0010972), cellular responses to stress (GO:0033554, GO:0016740), and protein binding (GO:0005515, GO:0044877, GO:0030674) (Table S12). At t_1_, common gained relationships were with extramodular genes associated with posttranscriptional regulation (GO:0140537, GO:0031123), (GO:0071007), development (GO:0032502, GO:0071840), and growth (GO:0051301, GO:0006260) (Table S13). Together, these results suggest a strong defence of transcriptional programs required for brain and face morphogenesis under heat stress via compensatory recruitment of additional genes to maintain a coordinated development program. Maintenance of intramodular coordination was supported by stronger extra‐modular relationships with genes related to negatively regulating transcription, a common stress response enacted to avoid proteotoxicity (Vihervaara et al. 2018), and post‐transcriptional regulation, which is a fast‐acting response to thermal challenge (Bock et al. 2020).

## 4 Conclusion

Our results demonstrate that embryonic resilience to heat stress emerges from coordinated regulation across multiple levels of biological organization. Rather than uniformly disrupting development, thermal stress triggers a hierarchical response: embryos preserve core regulatory modules essential for morphogenesis while dynamically suppressing transcription, modulating RNA flux, and recruiting auxiliary stress‐response pathways. This combination of robustness and flexibility enables embryonic tissues to maintain developmental pathways under conditions that would otherwise compromise viability. These findings advance a systems‐level framework for understanding developmental tolerance to environmental stress. Integrated regulatory mechanisms at vulnerable life stages are likely to play a central role in species persistence as climate change intensifies thermal variability and extremes. Identifying how these regulatory strategies vary across taxa, environments, and evolutionary histories is a critical next step toward predicting adaptive capacity and the trajectory of natural populations under global change.

Future work should determine whether these patterns are general across the species' range and whether they reflect functional mechanisms for maintaining morphogenesis. Necessary next steps include: (i) replication of this experiment using individuals across *A. sagrei'*s invasive and native range and among other oviparous ectothermic species to understand the universality of these observed patterns and test whether regulatory responses and the fitness costs of embryonic thermal stress have diverged during range expansion and colonization, (ii) protein‐level validation of key developmental regulators and newly recruited stress‐response genes to establish whether the transcription coordination observed here translates to coordinated changes in protein abundance and activity, (iii) perturbation experiments targeting candidate hub genes or stress‐response pathways through CRISPR‐mediated gene editing or pharmacological inhibition of proteostasis and RNA‐processing pathways could test whether recruitment of these genes is required to preserve forebrain and craniofacial development under thermal challenge. Such experiments would provide a critical mechanistic link between network rewiring, developmental robustness, and adaptive regulatory plasticity in warming environments.

## Author Contributions

S.C.C.‐S. and T.J.S. conceived of and conceptualized this study. R.H.W., I.S., and S.C.C.‐S. performed statistical analyses. T.J.S. conducted animal care and tissue collections. N.R. and I.S. performed all laboratory work. All results were visualized by R.H.W. and S.C.C.‐S. Supervision was provided by S.C.C.‐S. and T.J.S. The original draft of the manuscript was written by R.H.W. and S.C.C.‐S., then reviewed and edited by R.H.W., I.S., N.R., T.J.S., and S.C.C.‐S.

## Funding

This material is based upon work supported by the High Meadows Environmental Institute at Princeton University (awarded to S.C.C.‐S.), Alfred P. Sloan Foundation (Sloan Research Fellowship in Earth System Science, awarded to S.C.C.‐S.), and The U.S. National Science Foundation (DEB—2219279 awarded to S.C.C.‐S. and 1942250 to T.J.S.).

## Conflicts of Interest

The authors declare no conflicts of interest.

## Supporting information


**Figure S1:** Differentiated genes across incubation treatments and days of development. Venn diagrams illustrating the number of up‐ and down‐related genes shared among heat stress treatments and the percentage of unique genes in each overlap category.
**Figure S2:** Cluster dendrograms displaying patterns of correlated gene expression underpinning the embryonic brain and eye transcriptome under ideal (27°C, 24 h) and heat stressed (33°C, 24 h; 36°C, 8 h; 40°C, 1 h) incubation conditions. Each gene (*N* = 9626) is represented by an individual branch. Module colours identify groups of highly correlated genes.
**Figure S3:** Shared maintained and delayed or disrupted intramodular gene–gene relationships across incubation treatments and days of development. Venn diagrams illustrating the number of gene pairs shared among heat stress treatments and the percentage of unique pairs in each overlap category.
**Table S1:** Values and sources for key environmental parameters included in Niche Mapper's microclimate model for *A. sagrei* nest sites.
**Table S2:** Summary of principal components analyses of *A. sagrei* gene expression (*N* = 9626 genes) from embryonic telencephalon and eye tissue samples (*N* = 90) across four incubation treatments in the days following oviposition. Each column (PC1‐44) represents a principal component; only the first 44 of the 90 PCs are shown. > 70% of the variance in gene expression across samples is explained by the first two principle components.
**Table S3:** Parameter estimates from simple linear regression models comparing PC1 and PC2 values among heat treated samples to those under ideal incubation conditions (27°C, 24 h) immediately following (t_0_) and 24 h after (t_1_) heat stress.
**Table S4:** Driver terms for significant gene enrichment categories associated with differentially expressed genes shared across heat stress scenarios on the day of oviposition (t_0_) and 24 h later (t_1_).
**Table S5:** Function of post‐transcriptionally regulated genes under control (27°C, 24 h), chronic mild stress (33°C, 24 h), persistent moderate stress (36°C, 8 h), and heat shock (40°C, 1 h) incubation treatments identified through exon‐intron split analysis.
**Table S6:** Significant gene enrichment categories from an ordered query ranked by genes' module membership in the development‐associated module identified via WCGNA under control incubation conditions (27°C, 24 h).
**Table S7:** Driver terms in significant gene enrichment categories for the recovery‐associated module under persistent moderate stress incubation conditions (36°C, 8 h) identified through WGCNA.
**Table S8:** Driver terms in significant gene enrichment categories for the recovery‐associated modules under heat‐shock incubation conditions (40°C, 1 h) identified through WGCNA.
**Table S9:** Parameter estimates from simple linear regression models comparing intramodular coherence among heat stress scenarios to that observed under ideal incubation conditions (27°C, 24 h) immediately following (t_0_) and 24 h after (t_1_) heat stress.
**Table S10:** Driver terms in significant enrichment categories for genes represented in maintained intramodular gene–gene relationship shared across incubation conditions.
**Table S11:** Parameter estimates from simple linear regression models comparing extramodular coherence among heat stress scenarios to that observed under ideal incubation conditions (27°C, 24 h) immediately following (t_0_) and 24 h after (t_1_) heat stress.
**Table S12:** Driver terms in significant enrichment categories for genes most represented in gained extramodular gene–gene relationship shared across incubation conditions on the day of oviposition (t_0_).
**Table S13:** Driver terms in significant enrichment categories for genes most represented in gained extramodular gene–gene relationship shared across incubation conditions on the day after oviposition (t_1_).

## Data Availability

All data used in our analysis will be made publicly available in Dryad (https://doi.org/10.5061/dryad.59zw3r2mr) upon the acceptance of this manuscript for publication. Reviewers can access data at: http://datadryad.org/share/nqz8ZzczKVwBiqkTkTKWZKZjne‐TsBf2ZTAIK_5_zxM.
