# Supplementary material for: Regulatory Responses of Ectothermic Embryos to Predicted Heat Stresses Under Near‐Future Climate Change
Source: Mol Ecol. 2026 Jul 10;35(13):e70456. doi: 10.1111/mec.70456 (PMC13354517; doi:10.1111/mec.70456)
Supplement: Supplementary file 1 — Figure S1: Differentiated genes across incubation treatments and days of development. Venn diagrams illustrating the number of up‐ and down‐related genes shared among heat stress treatments and the percentage of unique genes in each overlap category. Figure S2: Cluster dendrograms displaying patterns of correlated gene expression underpinning the embryonic brain and eye transcriptome under ideal (27°C, 24 h) and heat stressed (33°C, 24 h; 36°C, 8 h; 40°C, 1 h) incubation conditions. Each gene (N = 9626) is represented by an individual branch. Module colours identify groups of highly correlated genes. Figure S3: Shared maintained and delayed or disrupted intramodular gene–gene relationships across incubation treatments and days of development. Venn diagrams illustrating the number of gene pairs shared among heat stress treatments and the percentage of unique pairs in each overlap category. Table S1: Values and sources for key environmental parameters included in Niche Mapper's microclimate model for A. sagrei nest sites. Table S2: Summary of principal components analyses of A. sagrei gene expression (N = 9626 genes) from embryonic telencephalon and eye tissue samples (N = 90) across four incubation treatments in the days following oviposition. Each column (PC1‐44) represents a principal component; only the first 44 of the 90 PCs are shown. > 70% of the variance in gene expression across samples is explained by the first two principle components. Table S3: Parameter estimates from simple linear regression models comparing PC1 and PC2 values among heat treated samples to those under ideal incubation conditions (27°C, 24 h) immediately following (t0) and 24 h after (t1) heat stress. Table S4: Driver terms for significant gene enrichment categories associated with differentially expressed genes shared across heat stress scenarios on the day of oviposition (t0) and 24 h later (t1). Table S5: Function of post‐transcriptionally regulated genes under control (27°C, 24 h), ch [file MEC-35-e70456-s001.docx]

***Supplementary Materials:* Regulatory responses of ectothermic embryos to predicted heat stresses under near-future climate change**

Reena H. Walker^1^, Ishani Sinha^1^, Nicholas Rochette^1^, Thomas J. Sanger^2^, & Shane C. Campbell-Staton^1*^

1. Department of Ecology & Evolutionary Biology, Princeton University, Princeton, NJ, USA.
2. Department of Biology, Loyola University Chicago, Chicago, IL, USA.

^*^ Correspondence may be addressed to: [hallie.walker.brown@gmail.com](mailto:hallie.walker.brown@gmail.com), [scampbellstaton@princeton.edu](mailto:scampbellstaton@princeton.edu)

**Supplemental Tables**

**Table S1.** **Values and sources for key environmental parameters included in Niche Mapper’s microclimate model for *A. sagrei* nest sites.**

| *Parameter* | *Source* | *Value* |
| --- | --- | --- |
| Soil thermal conductivity | Estimated from Abu-hamdeh & Reeder (2000) | 2.5 W/m^o^C |
| Substrate reflectivity | Estimated from Oguntunde et al (2006) | 20% |
| Substrate density | Estimated from Sharma (1977) | 1500 kg/m^3^ |
| Substrate specific heat | Estimated from Ren et al (2003) | 870 J/ kgK |
| Substrate longwave infrared emissions | Estimated from Sellers (1965) | 96% |
| Percent shade | Estimated from high lux nest sites reported in Sanger et al (2018) | 50 – 51% |
| Cloud cover | Sourced from EarthEnv datasets (Wilson & Jetz 2016) | 20 – 99% |
| Wind | Sourced from WorldClim 2 (Fick & Hijmans 2017) |  |
| Air Temperature | Sourced from WorldClim 2 historic and ACCESS-CM2 ssp370 downscaled future monthly climate data (Fick & Hijmans 2017) | 11 – 43^o^C |
|  |  |  |
| Elevation | Sourced from (Fick & Hijmans 2017) | -5 – 1602 m |
| Slope | Estimated from (Fick & Hijmans 2017) | 0 – 8.86^o^ |

**Table S2. Summary of principal components analyses of *A. sagrei* gene expression (N = 9626 genes) from embryonic telencephalon and eye tissue samples (N = 90) across four incubation treatments in the days following oviposition.** Each column (PC1-44) represents a principal component; only the first 44 of the 90 PCs are shown. >70% of the variance in gene expression across samples is explained by the first two principle components.

|  | *PC1* | *PC2* | *PC3* | *PC4* | *PC5* | *PC6* | *PC7* | *PC8* | *PC9* | *PC10* | *PC11* |
| --- | --- | --- | --- | --- | --- | --- | --- | --- | --- | --- | --- |
| *Standard deviation* | 12553.2 | 10663.8 | 4731.32 | 3857.85 | 3279.64 | 2412.379 | 2138.161 | 1879.256 | 1826.382 | 1683.729 | 1517.42 |
| *Proportion of variance* | 0.425 | 0.306 | 0.06 | 0.04 | 0.029 | 0.016 | 0.012 | 0.01 | 0.009 | 0.008 | 0.006 |
| *Cumulative proportion* | 0.425 | 0.731 | 0.791 | 0.831 | 0.86 | 0.876 | 0.888 | 0.898 | 0.907 | 0.914 | 0.921 |
|  | *PC12* | *PC13* | *PC14* | *PC15* | *PC16* | *PC17* | *PC18* | *PC19* | *PC20* | *PC21* | *PC22* |
| *Standard deviation* | 1355.11 | 1348.60 | 1241.14 | 1196.35 | 1149.48 | 1045.31 | 986.39 | 953.88 | 933.779 | 923.472 | 833.564 |
| *Proportion of variance* | 0.005 | 0.005 | 0.004 | 0.004 | 0.004 | 0.003 | 0.003 | 0.002 | 0.002 | 0.002 | 0.002 |
| *Cumulative proportion* | 0.926 | 0.931 | 0.935 | 0.939 | 0.942 | 0.945 | 0.948 | 0.95 | 0.952 | 0.955 | 0.957 |
|  | *PC23* | *PC24* | *PC25* | *PC26* | *PC27* | *PC28* | *PC29* | *PC30* | *PC31* | *PC32* | *PC33* |
| *Standard deviation* | 813.216 | 796.365 | 774.298 | 762.377 | 746.091 | 736.52 | 712.929 | 706.599 | 678.809 | 646.714 | 630.606 |
| *Proportion of variance* | 0.002 | 0.002 | 0.002 | 0.002 | 0.002 | 0.001 | 0.001 | 0.001 | 0.001 | 0.001 | 0.001 |
| *Cumulative proportion* | 0.958 | 0.96 | 0.962 | 0.963 | 0.965 | 0.966 | 0.968 | 0.969 | 0.97 | 0.971 | 0.972 |
|  | *PC34* | *PC35* | *PC36* | *PC37* | *PC38* | *PC39* | *PC40* | *PC41* | *PC42* | *PC43* | *PC44* |
| *Standard deviation* | 625.622 | 620.824 | 608.845 | 596.242 | 585.399 | 572.776 | 568.173 | 552.029 | 542.728 | 537.898 | 532.471 |
| *Proportion of variance* | 0.001 | 0.001 | 0.001 | 0.001 | 0.001 | 0.001 | 0.001 | 0.001 | 0.001 | 0.001 | 0.001 |
| *Cumulative proportion* | 0.973 | 0.975 | 0.976 | 0.976 | 0.977 | 0.978 | 0.979 | 0.98 | 0.981 | 0.982 | 0.982 |

**Table S3. Parameter estimates from simple linear regression models comparing PC1 and PC2 values among heat treated samples to those under ideal incubation conditions (27** **°C, 24 hrs) immediately following (t_0_) and 24 hours after (t_1_) heat stress.**

| **t_0_** | **Estimate** | **Standard Error** | **t value** | **Pr(>\|t\|)** |  |
| --- | --- | --- | --- | --- | --- |
| **PC1** |  |  |  |  |  |
| (Intercept) | -14,888.775 | 2,258.852 | -6.591 | 0.0000 | *** |
| 33°C, 24 hrs | 9,688.714 | 3,121.052 | 3.104 | 0.0035 | ** |
| 36°C, 8 hrs | 8,621.723 | 3,058.500 | 2.819 | 0.0074 | ** |
| 40°C, 1 hr | 10,657.959 | 3,058.500 | 3.485 | 0.0012 | ** |
| **PC2** |  |  |  |  |  |
| (Intercept) | -9,722.850 | 1,586.869 | -6.127 | 0.0000 | *** |
| 33°C, 24 hrs | 5,032.148 | 2,192.575 | 2.295 | 0.0269 | * |
| 36°C, 8 hrs | 15,916.024 | 2,148.631 | 7.408 | 0.0000 | *** |
| 40°C, 1 hr | 32,549.432 | 2,148.631 | 15.149 | 0.0000 | *** |
|  |  |  |  |  |  |
| **t_1_** | **Estimate** | **Standard Error** | **t value** | **Pr(>\|t\|)** |  |
| **PC1** |  |  |  |  |  |
| (Intercept) | 6,049.644 | 3,631.494 | 1.666 | 0.1034 |  |
| 33°C, 24 hrs | 2,691.566 | 5,135.708 | 0.524 | 0.6030 |  |
| 36°C, 8 hrs | 4,741.621 | 5,027.575 | 0.943 | 0.3511 |  |
| 40°C, 1 hr | -2,425.255 | 5,135.708 | -0.472 | 0.6393 |  |
| **PC2** |  |  |  |  |  |
| (Intercept) | -6,004.923 | 723.391 | -8.301 | 0.0000 | *** |
| 33°C, 24 hrs | 3,419.443 | 1,023.029 | 3.342 | 0.0018 | ** |
| 36°C, 8 hrs | 1,806.206 | 1,001.489 | 1.804 | 0.0787 | . |
| 40°C, 1 hr | 1,047.487 | 1,023.029 | 1.024 | 0.3119 |  |
| *Signif. codes: 0 <= '***' < 0.001 < '**' < 0.01 < '*' < 0.05* | | | | | |

**Table S4. Driver terms for significant gene enrichment categories associated with differentially expressed genes shared across heat stress scenarios on the day of oviposition (t_0_) and 24 hours later (t_1_).**

| **t_0_** | **upregulated** |  |  |  |  |
| --- | --- | --- | --- | --- | --- |
| **Source** | **GO term** | **GO name** | **GO term size** | **Query size** | **P** |
| GO:BP | GO:0007017 | microtubule-based process | 635 | 46 | 0.005 |
| GO:BP | GO:0044458 | motile cilium assembly | 44 | 46 | 0.017 |
| GO:BP | GO:0044782 | cilium organization | 263 | 46 | 0.026 |
| GO:BP | GO:0003351 | epithelial cilium movement involved in extracellular fluid movement | 19 | 46 | 0.045 |
| GO:MF | GO:0005515 | protein binding | 5,934 | 47 | 0.035 |
| **t_0_** | **downregulated** |  |  |  |  |
| **Source** | **GO term** | **GO name** | **GO term size** | **Query size** | **P** |
| GO:CC | GO:0005634 | nucleus | 4,899 | 18 | 0.026 |
| GO:CC | GO:0032993 | protein-DNA complex | 506 | 18 | 0.040 |
| GO:MF | GO:0042393 | histone binding | 148 | 16 | 0.003 |
| **t_1_** | **upregulated** |  |  |  |  |
| **Source** | **GO term** | **GO name** | **GO term size** | **Query size** | **P** |
| GO:CC | GO:0071005 | U2-type precatalytic spliceosome | 30 | 15 | 0.050 |
| **t_1_** | **downregulated** |  |  |  |  |
| **Source** | **GO term** | **GO name** | **GO term size** | **Query size** | **P** |
| GO:BP | GO:1900225 | regulation of NLRP3 inflammasome complex assembly | 16 | 5 | 0.006 |
| GO:MF | GO:0140545 | ATP-dependent protein disaggregase activity | 1 | 5 | 0.050 |

**Table S5. Function of post-transcriptionally regulated genes under control (27°C, 24 hr), chronic mild stress (33°C, 24 hr), persistent moderate stress (36°C, 8hr), and heat shock (40°C, 1 hr) incubation treatments identified through exon-intron split analysis.**

| **Treatment** | **ENSEMBL ID** | **Gene** | **Function** | **Citation** |
| --- | --- | --- | --- | --- |
| 27°C, 24 hr | ENSACAG00000009810 | *hnrnpl* | RNA metabolism | Mathew et al 2023 |
|  | ENSACAG00000014308 | *gadl1* | Neuron development | Winge et al 2015 |
|  | ENSACAG00000001219 | *mid1* | Brain development | Winter et al 2016 |
|  | ENSACAG00000003080 | *gtf2e2* | RNA metabolism | Kuschal et al 2016 |
| 33°C, 24 hr | ENSACAG00000010416 | *hnrnpl* | RNA metabolism | Li et al 2019 |
| 36°C, 8 hr | ENSACAG00000000798 | *wbp4* | RNA metabolism | Engal et al. 2023 |
|  | ENSACAG00000001831 | *gpm6b* | Brain development | Möbius et al 2008 |
|  | ENSACAG00000003511 | *rbp1* | Neural, eye development | Flach et al 2021 |
|  | ENSACAG00000003762 | *dtnbp1* | Brain development | Tognin et al 2011 |
|  | ENSACAG00000007429 | *sat1* | Brain development | Weiss et al 2003 |
|  | ENSACAG00000010416 | *hnrnpl* | RNA metabolism | Li et al 2019 |
|  | ENSACAG00000010642 | *ppp1r10* | DNA repair | White et al 2019 |
|  | ENSACAG00000010909 | *rad21* | Brain development | Pemberton et al 2007 |
|  | ENSACAG00000011593 | *rpn2* | Protein quality control | Sun et al 2020 |
|  | ENSACAG00000012576 | *qrich1* | Unfolded protein response | You et al 2021 |
|  | ENSACAG00000013621 | *caprin1* | Splicing, translation | Vu et al 2021 |
|  | ENSACAG00000015931 |  |  |  |
|  | ENSACAG00000016986 | *tial1* | Translation, apoptosis | Wang et al 2018 |
|  | ENSACAG00000001219 | *mid1* | Brain development | Winter et al 2016 |
|  | ENSACAG00000001842 | *ip6k2* | Mitigates neuronal death | Nagpal et al 2021 |
|  | ENSACAG00000005576 |  |  |  |
|  | ENSACAG00000009312 | *nipbl* | Brain development | van den Berg et al 2017 |
|  | ENSACAG00000013408 | *psmg2* | Unfolded protein response | Ebstein et al 2019 |
| 40°C, 1 hr | ENSACAG00000000465 |  |  |  |
|  | ENSACAG00000000798 | *wbp4* | RNA metabolism | Engal et al. 2023 |
|  | ENSACAG00000003550 | *pcca* | Metabolism | Campeau et al 2001 |
|  | ENSACAG00000005263 |  |  |  |
|  | ENSACAG00000006572 | *adgrl2* | Brain development | Donohue et al 2021 |
|  | ENSACAG00000007341 | *septin7* | Neuron development | Ageta-Ishihara 2021 |
|  | ENSACAG00000010909 | *rad21* | Brain development | Pemberton et al 2007 |
|  | ENSACAG00000010948 |  |  |  |
|  | ENSACAG00000013166 | *isy1* | RNA metabolism | Jaiswal et al 2020 |
|  | ENSACAG00000013836 | *kpna3* | Neuron development | Laitman et al 2017 |
|  | ENSACAG00000016197 | *rae1* | RNA metabolism | Pritchard et al. 1999 |
|  | ENSACAG00000016749 |  |  |  |
|  | ENSACAG00000016986 | *tial1* | Translation, apoptosis | Wang et al 2018 |
|  | ENSACAG00000001219 | *mid1* | Brain development | Winter et al 2016 |
|  | ENSACAG00000001842 | *ip6k2* | Mitigates neuronal death | Nagpal et al 2021 |

**Table S6. Significant gene enrichment categories from an ordered query ranked by genes’ module membership in the development-associated module identified via WCGNA under control incubation conditions (27** **°C, 24 hrs).**

| **Source** | **GO term** | **GO name** | **GO term size** | **Query size** | **P** |
| --- | --- | --- | --- | --- | --- |
| GO:BP | GO:1902850 | microtubule cytoskeleton organization involved in mitosis | 97 | 592 | 0.0001 |
| GO:BP | GO:0010001 | glial cell differentiation | 122 | 222 | 0.0004 |
| GO:BP | GO:0042063 | gliogenesis | 151 | 222 | 0.0006 |
| GO:BP | GO:0007052 | mitotic spindle organization | 80 | 592 | 0.0006 |
| GO:BP | GO:0048731 | system development | 2,204 | 232 | 0.0011 |
| GO:BP | GO:1903047 | mitotic cell cycle process | 425 | 601 | 0.0015 |
| GO:BP | GO:0007275 | multicellular organism development | 2,493 | 222 | 0.0015 |
| GO:BP | GO:0007010 | cytoskeleton organization | 945 | 625 | 0.0016 |
| GO:BP | GO:0007417 | central nervous system development | 459 | 222 | 0.0017 |
| GO:BP | GO:0051960 | regulation of nervous system development | 207 | 811 | 0.0030 |
| GO:BP | GO:0022008 | neurogenesis | 937 | 232 | 0.0035 |
| GO:BP | GO:1902850 | microtubule cytoskeleton organization involved in mitosis | 97 | 592 | 0.0001 |
| GO:BP | GO:0010001 | glial cell differentiation | 122 | 222 | 0.0004 |
| GO:BP | GO:0042063 | gliogenesis | 151 | 222 | 0.0006 |
| GO:BP | GO:0007052 | mitotic spindle organization | 80 | 592 | 0.0006 |
| GO:BP | GO:0048731 | system development | 2,204 | 232 | 0.0011 |
| GO:BP | GO:1903047 | mitotic cell cycle process | 425 | 601 | 0.0015 |
| GO:BP | GO:0007275 | multicellular organism development | 2,493 | 222 | 0.0015 |
| GO:BP | GO:0007010 | cytoskeleton organization | 945 | 625 | 0.0016 |
| GO:BP | GO:0007417 | central nervous system development | 459 | 222 | 0.0017 |
| GO:BP | GO:0051960 | regulation of nervous system development | 207 | 811 | 0.0030 |
| GO:BP | GO:0022008 | neurogenesis | 937 | 232 | 0.0035 |
| GO:BP | GO:0031175 | neuron projection development | 507 | 47 | 0.0046 |
| GO:BP | GO:0007399 | nervous system development | 1,297 | 222 | 0.0073 |
| GO:BP | GO:0007051 | spindle organization | 124 | 517 | 0.0079 |
| GO:BP | GO:0000278 | mitotic cell cycle | 513 | 601 | 0.0107 |
| GO:BP | GO:0061024 | membrane organization | 398 | 331 | 0.0117 |
| GO:BP | GO:0099175 | regulation of postsynapse organization | 54 | 204 | 0.0128 |
| GO:BP | GO:0050767 | regulation of neurogenesis | 166 | 811 | 0.0133 |
| GO:BP | GO:0048708 | astrocyte differentiation | 42 | 180 | 0.0163 |
| GO:BP | GO:0022402 | cell cycle process | 735 | 517 | 0.0177 |
| GO:BP | GO:0031145 | anaphase-promoting complex-dependent catabolic process | 17 | 497 | 0.0188 |
| GO:BP | GO:0048666 | neuron development | 595 | 47 | 0.0188 |
| GO:BP | GO:0048699 | generation of neurons | 803 | 267 | 0.0208 |
| GO:BP | GO:0021782 | glial cell development | 57 | 209 | 0.0216 |
| GO:BP | GO:0045838 | positive regulation of membrane potential | 4 | 223 | 0.0217 |
| GO:BP | GO:0045892 | negative regulation of DNA-templated transcription | 673 | 654 | 0.0241 |
| GO:BP | GO:1902679 | negative regulation of RNA biosynthetic process | 674 | 654 | 0.0251 |
| GO:BP | GO:0007049 | cell cycle | 903 | 642 | 0.0255 |
| GO:BP | GO:0006996 | organelle organization | 2,173 | 853 | 0.0261 |
| GO:BP | GO:0051128 | regulation of cellular component organization | 1,226 | 310 | 0.0301 |
| GO:BP | GO:0051301 | cell division | 279 | 570 | 0.0317 |
| GO:BP | GO:0016043 | cellular component organization | 4,166 | 849 | 0.0356 |
| GO:BP | GO:2000026 | regulation of multicellular organismal development | 670 | 21 | 0.0368 |
| GO:BP | GO:0051253 | negative regulation of RNA metabolic process | 738 | 654 | 0.0399 |
| GO:BP | GO:0051230 | spindle disassembly | 3 | 435 | 0.0410 |
| GO:BP | GO:0051228 | mitotic spindle disassembly | 3 | 435 | 0.0410 |
| GO:BP | GO:0006511 | ubiquitin-dependent protein catabolic process | 394 | 748 | 0.0427 |
| GO:BP | GO:0045934 | negative regulation of nucleobase-containing compound metabolic process | 795 | 654 | 0.0459 |
| GO:BP | GO:0071840 | cellular component organization or biogenesis | 4,316 | 849 | 0.0472 |
| GO:BP | GO:0016198 | axon choice point recognition | 3 | 47 | 0.0496 |
| GO:CC | GO:0005829 | cytosol | 2,271 | 849 | 0.0000 |
| GO:CC | GO:0099080 | supramolecular complex | 1,010 | 630 | 0.0000 |
| GO:CC | GO:0005737 | cytoplasm | 7,471 | 849 | 0.0000 |
| GO:CC | GO:0030426 | growth cone | 45 | 239 | 0.0001 |
| GO:CC | GO:0099513 | polymeric cytoskeletal fiber | 407 | 625 | 0.0001 |
| GO:CC | GO:1902494 | catalytic complex | 1,250 | 791 | 0.0002 |
| GO:CC | GO:0030427 | site of polarized growth | 49 | 239 | 0.0002 |
| GO:CC | GO:0005654 | nucleoplasm | 2,171 | 630 | 0.0004 |
| GO:CC | GO:0043228 | membraneless organelle | 3,623 | 653 | 0.0008 |
| GO:CC | GO:0043232 | intracellular membraneless organelle | 3,623 | 653 | 0.0008 |
| GO:CC | GO:0150034 | distal axon | 80 | 239 | 0.0010 |
| GO:CC | GO:0099512 | supramolecular fiber | 532 | 625 | 0.0010 |
| GO:CC | GO:0099081 | supramolecular polymer | 532 | 625 | 0.0010 |
| GO:CC | GO:0000785 | chromatin | 476 | 643 | 0.0016 |
| GO:CC | GO:0032991 | protein-containing complex | 4,297 | 682 | 0.0020 |
| GO:CC | GO:0005694 | chromosome | 836 | 643 | 0.0021 |
| GO:CC | GO:0036477 | somatodendritic compartment | 331 | 219 | 0.0047 |
| GO:CC | GO:0005583 | fibrillar collagen trimer | 6 | 157 | 0.0055 |
| GO:CC | GO:0098643 | fibrillar collagen complex | 6 | 157 | 0.0055 |
| GO:CC | GO:0005856 | cytoskeleton | 1,491 | 653 | 0.0064 |
| GO:CC | GO:0043025 | neuronal cell body | 158 | 324 | 0.0068 |
| GO:CC | GO:0005869 | dynactin complex | 6 | 579 | 0.0073 |
| GO:CC | GO:0140535 | intracellular protein-containing complex | 668 | 862 | 0.0087 |
| GO:CC | GO:0005874 | microtubule | 272 | 623 | 0.0088 |
| GO:CC | GO:0043005 | neuron projection | 618 | 11 | 0.0100 |
| GO:CC | GO:0022626 | cytosolic ribosome | 99 | 717 | 0.0113 |
| GO:CC | GO:0005838 | proteasome regulatory particle | 20 | 840 | 0.0144 |
| GO:CC | GO:0098794 | postsynapse | 341 | 219 | 0.0252 |
| GO:CC | GO:0044297 | cell body | 183 | 324 | 0.0311 |
| GO:CC | GO:0016363 | nuclear matrix | 43 | 102 | 0.0484 |
| GO:MF | GO:0005515 | protein binding | 5,839 | 594 | 0.0000 |
| GO:MF | GO:0008092 | cytoskeletal protein binding | 663 | 517 | 0.0000 |
| GO:MF | GO:0019899 | enzyme binding | 1,108 | 813 | 0.0001 |
| GO:MF | GO:0140657 | ATP-dependent activity | 484 | 533 | 0.0156 |
| GO:MF | GO:0031005 | filamin binding | 9 | 122 | 0.0171 |
| GO:MF | GO:0140658 | ATP-dependent chromatin remodeler activity | 14 | 499 | 0.0220 |
| GO:MF | GO:0061676 | importin-alpha family protein binding | 4 | 370 | 0.0234 |
| GO:MF | GO:0032452 | histone demethylase activity | 22 | 309 | 0.0251 |
| GO:MF | GO:0140457 | protein demethylase activity | 22 | 309 | 0.0251 |
| GO:MF | GO:0042802 | identical protein binding | 1,014 | 220 | 0.0259 |
| GO:MF | GO:0019903 | protein phosphatase binding | 72 | 300 | 0.0278 |

**Table S7. Driver terms in significant gene enrichment categories for the recovery-associated module under persistent moderate stress incubation conditions (36°C, 8 hrs) identified through WGCNA.**

| **Source** | **GO term** | **GO name** | **GO size** | **Query size** | **P** |
| --- | --- | --- | --- | --- | --- |
| GO:BP | GO:0006457 | protein folding | 148 | 308 | 0.0000 |
| GO:BP | GO:0006996 | organelle organization | 2,209 | 308 | 0.0000 |
| GO:BP | GO:0033554 | cellular response to stress | 980 | 308 | 0.0000 |
| GO:BP | GO:0007049 | cell cycle | 931 | 308 | 0.0009 |
| GO:BP | GO:0009057 | macromolecule catabolic process | 817 | 308 | 0.0038 |
| GO:BP | GO:1903320 | regulation of protein modification by small protein conjugation or removal | 112 | 308 | 0.0052 |
| GO:BP | GO:0006275 | regulation of DNA replication | 64 | 308 | 0.0093 |
| GO:BP | GO:0072708 | response to sorbitol | 3 | 308 | 0.0127 |
| GO:BP | GO:0050821 | protein stabilization | 84 | 308 | 0.0132 |
| GO:CC | GO:0005622 | intracellular anatomical structure | 10,265 | 323 | 0.0000 |
| GO:CC | GO:0101031 | protein folding chaperone complex | 23 | 323 | 0.0319 |
| GO:MF | GO:0051082 | unfolded protein binding | 73 | 305 | 0.0000 |
| GO:MF | GO:0140662 | ATP-dependent protein folding chaperone | 30 | 305 | 0.0000 |
| GO:MF | GO:0060590 | ATPase regulator activity | 31 | 305 | 0.0000 |
| GO:MF | GO:0044389 | ubiquitin-like protein ligase binding | 174 | 305 | 0.0006 |
| GO:MF | GO:0031072 | heat shock protein binding | 81 | 305 | 0.0014 |
| GO:MF | GO:0051087 | protein-folding chaperone binding | 78 | 305 | 0.0074 |
| GO:MF | GO:1901363 | heterocyclic compound binding | 1,854 | 305 | 0.0094 |
| GO:MF | GO:0005524 | ATP binding | 1,181 | 305 | 0.0395 |

**Table S8. Driver terms in significant gene enrichment categories for the recovery-associated modules under heat-shock incubation conditions (40°C, 1 hrs) identified through WGCNA.**

| **Module19** |  |  |  |  |  |
| --- | --- | --- | --- | --- | --- |
| **source** | **GO term** | **GO name** | **GO term size** | **Query size** | **P** |
| GO:BP | GO:0006457 | protein folding | 148 | 60 | 0.0000 |
| GO:BP | GO:0009408 | response to heat | 41 | 60 | 0.0009 |
| GO:CC | GO:0005829 | cytosol | 2,157 | 68 | 0.0073 |
| GO:CC | GO:0043229 | intracellular organelle | 8,870 | 68 | 0.0212 |
| GO:CC | GO:0005654 | nucleoplasm | 2,013 | 68 | 0.0237 |
| GO:CC | GO:0030018 | Z disc | 59 | 68 | 0.0399 |
| GO:MF | GO:0044183 | protein folding chaperone | 44 | 64 | 0.0000 |
| GO:MF | GO:0051082 | unfolded protein binding | 73 | 64 | 0.0000 |
| GO:MF | GO:0031072 | heat shock protein binding | 81 | 64 | 0.0005 |
| GO:MF | GO:0005488 | binding | 11,727 | 64 | 0.0012 |
| GO:MF | GO:0060590 | ATPase regulator activity | 31 | 64 | 0.0037 |
| GO:MF | GO:0031418 | L-ascorbic acid binding | 14 | 64 | 0.0116 |
| GO:MF | GO:0097718 | disordered domain specific binding | 16 | 64 | 0.0178 |
| GO:MF | GO:0030554 | adenyl nucleotide binding | 1,299 | 64 | 0.0252 |
| **Module6** |  |  |  |  |  |
| **source** | **GO term** | **GO name** | **GO term size** | **Query size** | **P** |
| GO:MF | GO:0016250 | N-sulfoglucosamine sulfohydrolase activity | 1 | 1 | 0.0205 |
| GO:MF | GO:0016826 | hydrolase activity, acting on acid sulfur-nitrogen bonds | 1 | 1 | 0.0205 |
| **Module16** |  |  |  |  |  |
| **source** | **GO term** | **GO name** | **GO term size** | **Query size** | **P** |
| GO:BP | GO:0170036 | import into the mitochondrion | 36 | 65 | 0.0241 |
| GO:BP | GO:0070124 | mitochondrial translational initiation | 2 | 84 | 0.0429 |
| KEGG | KEGG:00071 | Fatty acid degradation | 26 | 68 | 0.0494 |
| **Module24** |  |  |  |  |  |
| **source** | **GO term** | **GO name** | **GO term size** | **Query size** | **P** |
| GO:MF | GO:0030547 | signaling receptor inhibitor activity | 10 | 26 | 0.0491 |
| **Module7** |  |  |  |  |  |
| **source** | **GO term** | **GO name** | **GO term size** | **Query size** | **P** |
| GO:CC | GO:0140535 | intracellular protein-containing complex | 676 | 43 | 0.0009 |
| GO:CC | GO:0000151 | ubiquitin ligase complex | 217 | 43 | 0.0115 |
| GO:MF | GO:0016796 | exonuclease activity, active with either ribo- or deoxyribonucleic acids and producing 5'-phosphomonoesters | 45 | 153 | 0.0277 |

**Table S9. Parameter estimates from simple linear regression models comparing intramodular coherence among heat stress scenarios to that observed under ideal incubation conditions (27** **°C, 24 hrs) immediately following (t_0_) and 24 hours after (t_1_) heat stress.**

|  | **Estimate** | **Standard Error** | **t value** | **Pr(>\|t\|)** |  |
| --- | --- | --- | --- | --- | --- |
| **t_0_** |  |  |  |  |  |
| (Intercept) | 0.141 | 0.00041 | 341.48 | < 0.001 | *** |
| 33°C, 24 hrs | 0.085 | 0.00058 | 144.82 | < 0.001 | *** |
| 36°C, 8 hrs | 0.035 | 0.00058 | 59.88 | < 0.001 | *** |
| 40°C, 1 hr | 0.133 | 0.00058 | 228.28 | < 0.001 | *** |
| **t_1_** |  |  |  |  |  |
| (Intercept) | 0.417 | 0.00037 | 1103.43 | < 0.001 | *** |
| 33°C, 24 hrs | 0.014 | 0.00053 | 26.66 | < 0.001 | *** |
| 36°C, 8 hrs | -0.129 | 0.00053 | -240.57 | < 0.001 | *** |
| 40°C, 1 hr | -0.106 | 0.00053 | -197.56 | < 0.001 | *** |
|  |  | *Signif. codes: 0 <= '***' < 0.001 < '**' < 0.01 < '*' < 0.05* | | | |

**Table S10. Driver terms in significant enrichment categories for genes represented in maintained intramodular gene-gene relationship shared across incubation conditions.**

| **Source** | **GO term** | **GO name** | **GO size** | **Query size** | **P** |
| --- | --- | --- | --- | --- | --- |
| GO:BP | GO:0071840 | cellular component organization or biogenesis | 4,295 | 770 | 0.0000 |
| GO:BP | GO:0048731 | system development | 2,006 | 770 | 0.0000 |
| GO:BP | GO:0051641 | cellular localization | 1,994 | 770 | 0.0000 |
| GO:BP | GO:0006518 | peptide metabolic process | 591 | 770 | 0.0001 |
| GO:BP | GO:0007049 | cell cycle | 931 | 770 | 0.0001 |
| GO:BP | GO:0048523 | negative regulation of cellular process | 2,699 | 770 | 0.0002 |
| GO:BP | GO:0044271 | cellular nitrogen compound biosynthetic process | 3,078 | 770 | 0.0002 |
| GO:BP | GO:0045892 | negative regulation of DNA-templated transcription | 655 | 770 | 0.0002 |
| GO:BP | GO:0043161 | proteasome-mediated ubiquitin-dependent protein catabolic process | 255 | 770 | 0.0018 |
| GO:BP | GO:0007017 | microtubule-based process | 635 | 770 | 0.0074 |
| GO:BP | GO:0040007 | growth | 430 | 770 | 0.0091 |
| GO:BP | GO:0006338 | chromatin remodeling | 365 | 770 | 0.0100 |
| GO:BP | GO:0018130 | heterocycle biosynthetic process | 2,538 | 770 | 0.0261 |
| GO:BP | GO:0019438 | aromatic compound biosynthetic process | 2,543 | 770 | 0.0293 |
| GO:BP | GO:0030162 | regulation of proteolysis | 185 | 770 | 0.0490 |
| GO:CC | GO:0005737 | cytoplasm | 7,057 | 811 | 0.0000 |
| GO:CC | GO:0032991 | protein-containing complex | 4,282 | 811 | 0.0000 |
| GO:CC | GO:0034399 | nuclear periphery | 57 | 811 | 0.0193 |
| GO:MF | GO:0005515 | protein binding | 5,934 | 764 | 0.0000 |
| GO:MF | GO:0003712 | transcription coregulator activity | 313 | 764 | 0.0047 |
| GO:MF | GO:0030554 | adenyl nucleotide binding | 1,299 | 764 | 0.0105 |
| GO:MF | GO:0019903 | protein phosphatase binding | 70 | 764 | 0.0125 |
| GO:MF | GO:0003727 | single-stranded RNA binding | 54 | 764 | 0.0210 |
| GO:MF | GO:0061629 | RNA polymerase II-specific DNA-binding transcription factor binding | 182 | 764 | 0.0347 |

**Table S11. Parameter estimates from simple linear regression models comparing extramodular coherence among heat stress scenarios to that observed under ideal incubation conditions (27** **°C, 24 hrs) immediately following (t_0_) and 24 hours after (t_1_) heat stress.**

|  | **Estimate** | **Standard Error** | **t value** | **Pr(>\|t\|)** |  |
| --- | --- | --- | --- | --- | --- |
| **t_0_** |  |  |  |  |  |
| (Intercept) | 0.064  0.1 | 0.00032 | 201.2 | < 0.001 | *** |
| 33°C, 24 hrs | 0.109 | 0.00045 | 241.8 | < 0.001 | *** |
| 36°C, 8 hrs | 0.056 | 0.00045 | 125.1 | < 0.001 | *** |
| 40°C, 1 hr | 0.127 | 0.00045 | 282.7 | < 0.001 | *** |
| **t_1_** |  |  |  |  |  |
| (Intercept) | 0.211 | 0.00030 | 704.8 | < 0.001 | *** |
| 33°C, 24 hrs | 0.147 | 0.00042 | 347.6 | < 0.001 | *** |
| 36°C, 8 hrs | -0.019 | 0.00042 | -43.91 | < 0.001 | *** |
| 40°C, 1 hr | 0.056 | 0.00042 | 131.6 | < 0.001 | *** |
|  |  | *Signif. codes: 0 <= '***' < 0.001 < '**' < 0.01 < '*' < 0.05* | | | |

**Table S12. Driver terms in significant enrichment categories for genes most represented in gained extramodular gene-gene relationship shared across incubation conditions on the day of oviposition (t_0_).**

| **t_0_** |  |  |  |  |  |
| --- | --- | --- | --- | --- | --- |
| **Source** | **GO term** | **GO name** | **GO size** | **Query size** | **P** |
| GO:BP | GO:0071840 | cellular component organization or biogenesis | 4,295 | 1,052 | 0.0000 |
| GO:BP | GO:0048519 | negative regulation of biological process | 2,842 | 1,052 | 0.0000 |
| GO:BP | GO:0034660 | ncRNA metabolic process | 427 | 1,052 | 0.0005 |
| GO:BP | GO:0009057 | macromolecule catabolic process | 817 | 1,052 | 0.0034 |
| GO:BP | GO:0031570 | DNA integrity checkpoint signaling | 79 | 1,052 | 0.0036 |
| GO:BP | GO:0033554 | cellular response to stress | 980 | 1,052 | 0.0126 |
| GO:BP | GO:0010972 | negative regulation of G2/M transition of mitotic cell cycle | 39 | 1,052 | 0.0192 |
| GO:BP | GO:0051641 | cellular localization | 1,994 | 1,052 | 0.0210 |
| GO:BP | GO:1901564 | organonitrogen compound metabolic process | 4,018 | 1,052 | 0.0358 |
| GO:BP | GO:0051603 | proteolysis involved in protein catabolic process | 455 | 1,052 | 0.0358 |
| GO:BP | GO:0006325 | chromatin organization | 425 | 1,052 | 0.0398 |
| GO:BP | GO:0010948 | negative regulation of cell cycle process | 169 | 1,052 | 0.0413 |
| GO:BP | GO:0009058 | biosynthetic process | 5,583 | 1,052 | 0.0445 |
| GO:BP | GO:0036465 | synaptic vesicle recycling | 49 | 1,052 | 0.0491 |
| GO:CC | GO:0005622 | intracellular anatomical structure | 10,265 | 1,095 | 0.0000 |
| GO:CC | GO:1902494 | catalytic complex | 1,276 | 1,095 | 0.0000 |
| GO:MF | GO:0005515 | protein binding | 5,934 | 1,006 | 0.0000 |
| GO:MF | GO:0030554 | adenyl nucleotide binding | 1,299 | 1,006 | 0.0004 |
| GO:MF | GO:0140537 | transcription regulator activator activity | 5 | 1,006 | 0.0005 |
| GO:MF | GO:0044877 | protein-containing complex binding | 966 | 1,006 | 0.0005 |
| GO:MF | GO:0016740 | transferase activity | 1,970 | 1,006 | 0.0036 |
| GO:MF | GO:0030674 | protein-macromolecule adaptor activity | 544 | 1,006 | 0.0130 |

**Table S13. Driver terms in significant enrichment categories for genes most represented in gained extramodular gene-gene relationship shared across incubation conditions on the day after oviposition (t_1_).**

| **t_1_** |  |  |  |  |  |
| --- | --- | --- | --- | --- | --- |
| **Source** | **GO term** | **GO name** | **GO size** | **Query size** | **P** |
| GO:BP | GO:0071840 | cellular component organization or biogenesis | 4,295 | 2,004 | 0.0000 |
| GO:BP | GO:0006260 | DNA replication | 172 | 2,004 | 0.0000 |
| GO:BP | GO:0034660 | ncRNA metabolic process | 427 | 2,004 | 0.0000 |
| GO:BP | GO:0032502 | developmental process | 3,196 | 2,004 | 0.0000 |
| GO:BP | GO:0006793 | phosphorus metabolic process | 1,554 | 2,004 | 0.0012 |
| GO:BP | GO:0042254 | ribosome biogenesis | 213 | 2,004 | 0.0017 |
| GO:BP | GO:0006368 | transcription elongation by RNA polymerase II | 48 | 2,004 | 0.0022 |
| GO:BP | GO:0051301 | cell division | 250 | 2,004 | 0.0035 |
| GO:BP | GO:0009058 | biosynthetic process | 5,583 | 2,004 | 0.0050 |
| GO:BP | GO:0035556 | intracellular signal transduction | 1,535 | 2,004 | 0.0051 |
| GO:BP | GO:0031123 | RNA 3'-end processing | 63 | 2,004 | 0.0058 |
| GO:BP | GO:1903311 | regulation of mRNA metabolic process | 179 | 2,004 | 0.0322 |
| GO:BP | GO:0006839 | mitochondrial transport | 103 | 2,004 | 0.0401 |
| GO:BP | GO:0002181 | cytoplasmic translation | 70 | 2,004 | 0.0424 |
| GO:CC | GO:0005622 | intracellular anatomical structure | 10,265 | 2,057 | 0.0000 |
| GO:CC | GO:0030139 | endocytic vesicle | 97 | 2,057 | 0.0182 |
| GO:CC | GO:0099080 | supramolecular complex | 740 | 2,057 | 0.0309 |
| GO:CC | GO:0005681 | spliceosomal complex | 134 | 2,057 | 0.0315 |
| GO:CC | GO:0071007 | U2-type catalytic step 2 spliceosome | 20 | 2,057 | 0.0459 |
| GO:MF | GO:0005515 | protein binding | 5,934 | 1,954 | 0.0000 |
| GO:MF | GO:0003824 | catalytic activity | 5,607 | 1,954 | 0.0000 |
| GO:MF | GO:0060090 | molecular adaptor activity | 633 | 1,954 | 0.0001 |
| GO:MF | GO:0140537 | transcription regulator activator activity | 5 | 1,954 | 0.0123 |
| GO:MF | GO:0060590 | ATPase regulator activity | 31 | 1,954 | 0.0164 |
| GO:MF | GO:0019783 | ubiquitin-like protein peptidase activity | 102 | 1,954 | 0.0402 |
| GO:MF | GO:0016818 | hydrolase activity, acting on acid anhydrides, in phosphorus-containing anhydrides | 593 | 1,954 | 0.0482 |

**Supplemental Figures**

**
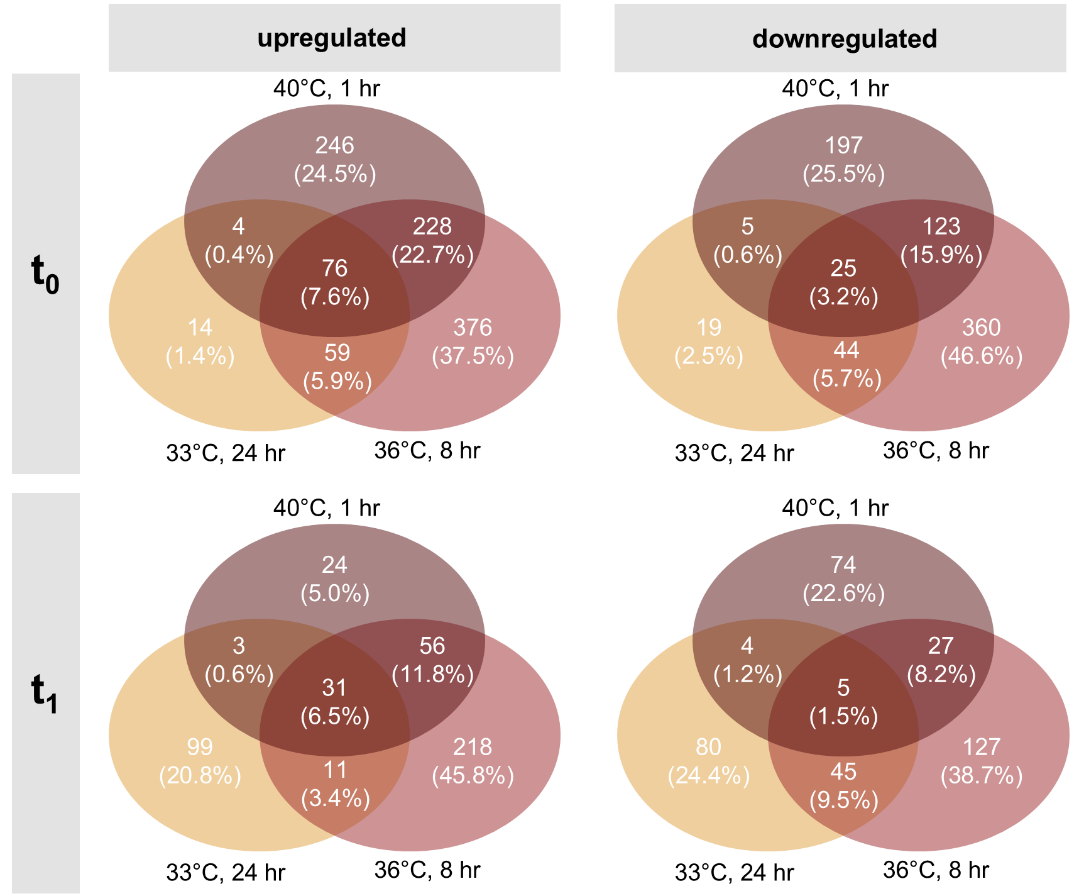
**

**Figure S1 | Differentiated genes across incubation treatments and days of development.** Venn diagrams illustrating the number of up- and down-related genes shared among heat stress treatments and the percentage of unique genes in each overlap category.

**
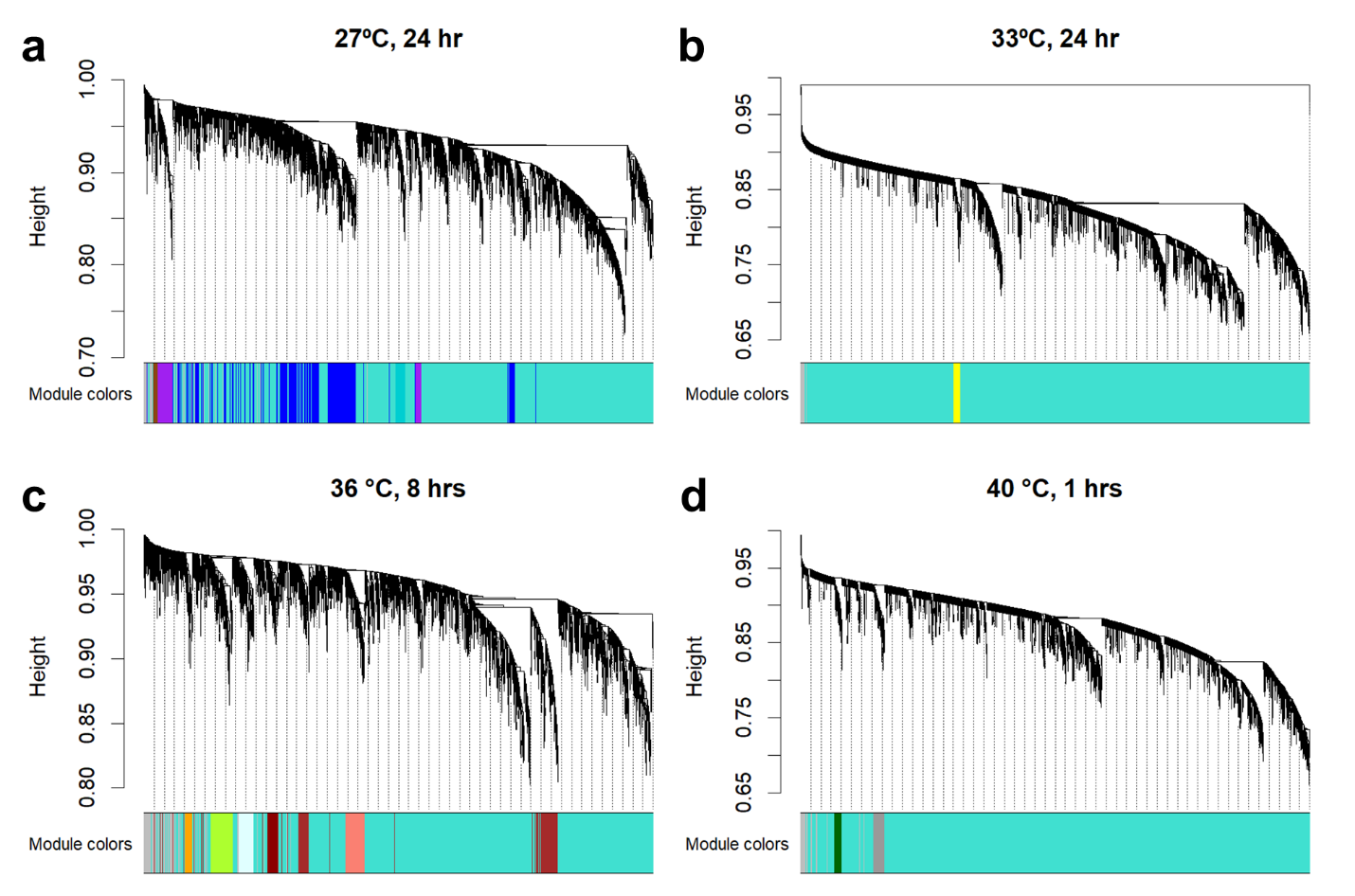
**

**Figure S2 | Cluster dendrograms displaying patterns of correlated gene expression underpinning the embryonic brain and eye transcriptome under ideal (27ºC, 24 hr) and heat stressed (33ºC, 24 hr; 36ºC, 8 hr; 40ºC, 1 hr) incubation conditions.** Each gene (N = 9626) is represented by an individual branch. Module colors identify groups of highly correlated genes.

**Figure S3 | Shared maintained and delayed or disrupted intramodular gene-gene relationships across incubation treatments and days of development.** Venn diagrams illustrating the number of gene pairs shared among heat stress treatments and the percentage of unique pairs in each overlap category.


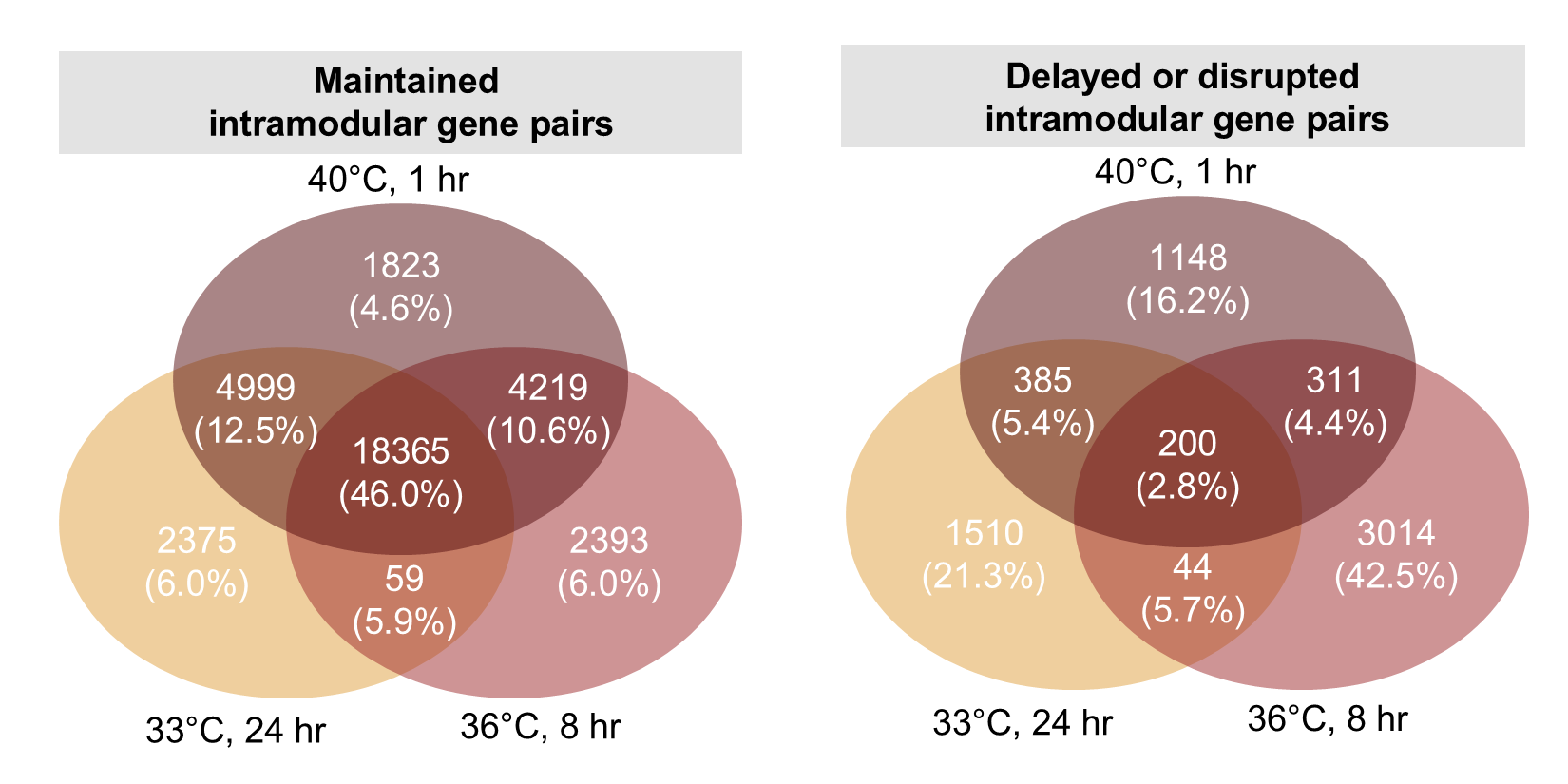


**References**

Abu-hamdeh, N. H., & Reeder, R. C. 2000. Soil thermal conductivity: Effects of density, moisture, salt concentration, and organic matter. *Soil Sci. Soc. Am*. **64**:1285-1290.

Ageta-Ishihara, N., & Kinoshita, M. 2021. Developmental and postdevelopmental roles of septins in the brain. *Neuroscience Research* **170**:6-12.

Campeau, E., Desviat, L.R., Leclerc, D., Wu, X., Pérez, B., Ugarte, M., Gravel, R.A. 2001. Structure of the *PCCA* gene and distribution of mutations causing propionic acidemia. *Molecular Genetics and Metabolism* **74**:238-247.

Donohue, J.D., et al. 2021. Parahippocampal latrophilin-2 (ADGRL2) expression controls topographical presubiculum to entorhinal cortex circuit connectivity. *Cell Reports* **37**:110031.

Ebstein, F., Harlowe, M.C., Studencka-Turski, M., & Krüger, E. 2019. Contribution of the unfolded protein response (UPR) to the pathogenesis of proteasome-associated autoinflammatory syndromes (PRASS). *Frontiers in Immunology* **25**:1-14.

Engal, E., et al. 2023. Bi-allelic loss-of-function variants in *WBP4*, encoding a spliceosome protein, result in a variable neurodevelopmental syndrome. *American Journal of Human Genetics* **110**:2112-2119.

Flach, H., et al. 2021. Retinol binding protein 1 affects *Xenopus* anterior neural development via *all-trans* retinoic acid signaling. *Developmental Dynamics* **250**:1096-1112.

Jaiswal, A.S., et al. 2020. The splicing component *ISY1* regulates *APE1* in base excision repair. *DNA Repair* **86**:102769.

Kuchal, C., et al. 2016. *GTF2E2* mutations destabilize the general transcription factor complex TFIIE in individuals with DNA repair-proficient trichothiodystrophy. *American Journal of Human Genetics* **98**:627-642.

Laitman, B.M., Mariani, J.N., Zhang, C., Sawai, S., & John, G.R. 2017. Karyopherin alpha proteins regulate oligodendrocyte differentiation. *PLoS ONE* **12**:e0170477.

Li, R.Z., Hou, J., Wei, Y., Luo, X., Ye, Y., & Zhang, Y. 2019. *hnRNPDL* extensively regulates transcription and alternative splicing. *Gene* **687**:125-134.

Mathew, B., et al. 2023. *hnRNPL* expression dynamics in the embryo and placenta. *Gene Expression
Patterns* **48**:119319.

Möbius, W., Patzig, J., Nave, K.A., & Werner, H.B. 2008. Phylogeny of proteolipid proteins: divergence, constraints, and the evolution of novel functions in myelination and neuroprotection. *Neuron Glia Biology* **4**:111-127.

Nagpal, L. Kornberg, M.D., Albacarys, L.K., & Snyder, S.H. 2021. Inositol hexakisphosphate kinase-2 determines cellular energy dynamics by regulating creatine kinase-B. *PNAS* **118**:e2020695118.

Ogunteunde, P. G., Ajayi, A. E., & van de Giesen, N. 2006. Tillage and surface moisture effects on bare-soil albedo of a tropical loamy sand. *Soil and Tillage Research* **85**:107-114. doi: 10.1016/j.still.2004.12.009.

Pemberton, H.N. et al. 2007. Separase, secruin, and Rad21 in neural cell growth. *Journal of Cellular Physiology* **213**:45-53.

Prichard, C.E.J., Fornerod, M., Kasper, L.H., & van Deursen, J.M.A. 1999. *RAE1* is a shuttling mRNA export factor that binds to a GLEBS-like NUP98 motif at the nuclear pore complex through multiple domains. *Journal of Cell Biology* **145**:237-254.

Ren, T., Ochsner, T. E. , Horton, R., & Ju, Z. 2003. Heat-pulse method for soil water content measurement: influence of the specific heat of the soil solids. *Soil Sci. Soc. Am.* **67**:1631-1634.

Sanger et al. (2021). Environmental thermal stress induces neuronal cell death and developmental malformations in reptiles. *Integrative Organismal Biology* **3**:1-18. doi: 10.1093/iob/obab033.

Sellers, W. D. *Physical climatology* (University of Chicago Press, 1965).

Sharma, V. *Environmental and Engineering Geophysics*. (Cambridge University Press, 1997).

Sun, J., et al. 2020. RPN2 is targeted by miR-181c and mediates glioma progression and temozolomide sensitivity via the wnt/β-signaling pathway. *Cell Death & Disease* **11**:1-15.

Tognin, S., Viding, E., McCrory, E.J., Taylor, L., O’Donovan, M.C., McGuire, P., & Mechelli, A. 2011. Effects of *DTNBP1* genotype on brain development in children. *Journal of Child Psychology and Phychiatry* **52**:1287-1297.

van den Berg, D.L.C., et al. 2017. *NIPBL* interacts with *Zfp609* and the integrator complex to regulate cortical neuron migration. *Neuron* **93**:348-361.

Vu, L., et al. 2021. Defining the Caprin-1 interactome in unstressed and stressed conditions. *Journal of Proteome Research* **20**:3165-3178.

Wang, Q., Yu, H., Yu, H., Ma, M., Ma, Y., & Li, R. 2018. miR-223-3p/TIAL1 interaction is involved in the mechanisms associated with the neuroprotective effects of dexmedetomidine on hippocampal neuronal cells in vitro. *Molecular Medicine Reports* **19**:805-812.

Weiss, M.D., Derazi, S., Rossignol, C., Varoqui, H., Erickson, J.D., Kilberg, M.S., & Anderson, K.J. 2003. Ontology of the neutral amino acid transporter *SAT1/ATA1* in rat brain. *Developmental Brain Research* **143**:515-159.

White, C.R., Dungan, M., & Carrithers, M.D. 2019. Activation of human macrophage sodium channels regulates RNA processing to increase expression of the DNA repair protein *PPP1R10*. *Immunobiology* **224**:80-93.

Wilson, AM & Jetz, W. 2016. Remotely sensed high-resolution global cloud dynamics for predicting ecosystem and biodiversity distributions. *PLoS Biol* **14**:e1002415. doi: 10.1371/journal.pbio.1002415.

Winge, I., Teigen, K., Fossbakk, A., Machootchi, E., Kleppe, R., Sköldberg, F., Kämpe, O., & Haavik, J. 2015. Mammalian CSAD and GADL1 have distinct biochemical properties and patterns of brain expression. *Neurochemistry International* **90**:173-184.

Winter, J., Basilicata, M.F., Stemmler, M.P., & Krausss, S. 2016. The *MID1* protein is a central player during development and in disease. *Frontiers in Bioscience* **21**:664-682.

You, K., et al. 2021. QRICH1 dictates the outcome of ER stress through transcriptional control of proteostasis. *Science* **371**:6524.
